# Records of three mammal tick species parasitizing an atypical host, the multi-ocellated racerunner lizard, in arid regions of Xinjiang, China

**DOI:** 10.1186/s13071-021-04639-z

**Published:** 2021-03-04

**Authors:** Qi Zhou, Jiao Li, Xianguang Guo, Jinlong Liu, Qi Song, Xiong Gong, Han Chen, Jianhui Zhang, Jinlei He, Zhiwan Zheng, Dali Chen, Jianping Chen

**Affiliations:** 1 Department of Pathogenic Biology, School of Basic Medical Sciences and Forensic Medicine, Sichuan University, Chengdu, West China China; 2 Chengdu Institute of Biology, Chinese Academy of Sciences, Chengdu, Sichuan China; 3 Animal Disease Prevention and Food Safety Key Laboratory of Sichuan Province, Sichuan University, Chengdu, Sichuan China

**Keywords:** Tick, Lizard, Xinjiang, *Hyalomma asiaticum*, *Rhipicephalus turanicus*, *Haemaphysalis sulcata*, *Eremias multiocellata*

## Abstract

**Background:**

Ticks are ectoparasites that feed on blood of a broad taxonomical range of terrestrial and flying vertebrates and are distributed across a wide range of environmental settings. To date, the species identity, diversity, and relationships among the ticks on lizards in China have been poorly understood.

**Methods:**

In this study, 30 ticks, collected from the multi-ocellated racerunner (*Eremias multiocellata*) lizard in the Tarim Basin and adjacent Yanqi Basin of the Xinjiang Uygur Autonomous Region in China, were identified by morphological observation and confirmed by DNA-based techniques. The mitochondrially encoded *12S rRNA*, *16S rRNA*, and *COI* gene fragments of ticks were amplified and sequenced. To understand the genetic polymorphisms, 47 ticks collected from hedgehogs and 1 from brushwood in the Tarim Basin were also included. Species identification was based on both morphological and molecular characters. The median-joining network approach was used to evaluate the intraspecific genealogies of the ticks and their relatedness with the geographical origin or hosts.

**Results:**

The sequence similarity analysis confirmed that the 30 ticks belong to three genera and three species including 11 individuals of *Hyalomma asiaticum*, 3 of *Rhipicephalus turanicus*, and 16 of *Haemaphysalis sulcata*. A network approach revealed paraphyletic populations of *R. turanicus* and *Hy. asiaticum* at the intraspecies level regarding geographical origin and low host specificity. For *R. turanicus* and *Hy. asiaticum*, common ancestry was observed between *COI* sequences from lizards and other sequence types from different hosts and countries.

**Conclusions:**

To our knowledge, our study is the first to conduct a molecular survey of ticks from lizards in the arid regions of Xinjiang, China. *Eremias multiocellata* is an atypical host of the three tick species. Notably, two species of ticks, *Hy. asiaticum* and *R. turanicus*, have been collected and identified from lizards in China for the first time. Star-like networks suggest both of them might have experienced recent population expansion. The discoveries are closely related to the geographical environments in Xinjiang and will provide information for the control of ticks and tick-borne pathogens in Northwest China.
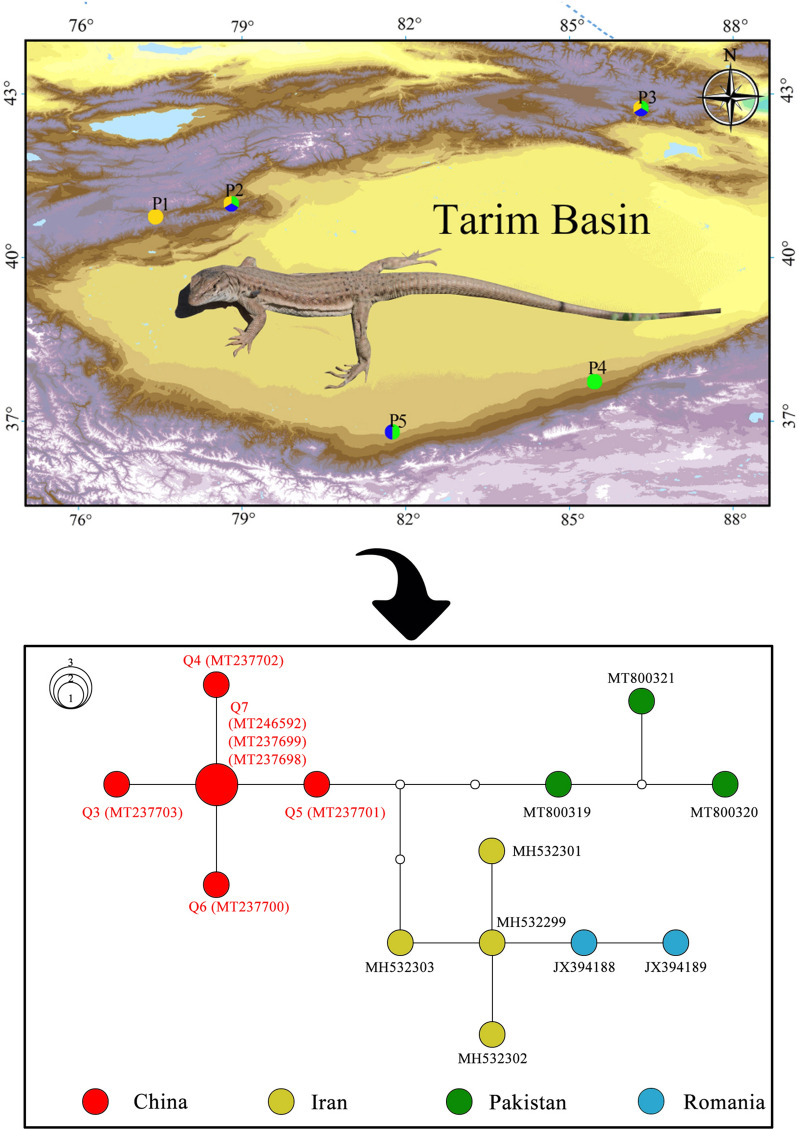

## Background

Ticks are obligate hematophagous ectoparasites of vertebrate animals, including amphibians, reptiles, birds, and mammals [1, 2]. Ticks can be found in areas around the world, ranging from the Arctic to tropical regions, and are considered significant vectors of diseases in humans, livestock, and wildlife [2]. Extant ticks comprise three families: Argasidae, Ixodidae, and Nuttalliellidae [3]. In China, ticks have high species diversity (~ 125 species) and have been classified into two families: Argasidae and Ixodidae [4, 5].

The Xinjiang Uygur Autonomous Region, located in the northwest of China, covers more than one sixth of the country’s territory. Large areas of mountains, deserts, and other additional characteristics constitute the particular landscape of Xinjiang [6]. Additionally, this region is mainly occupied with animal husbandry. Both the landscape and livestock population contribute to the survival of ticks. More than 40 tick species have been confirmed to be distributed in Xinjiang, constituting about one third of the species found in China, and most of the parasitifers are livestock [7].

Generally, wild animals serve as a huge and often unknown reservoir of hosts for zoonotic disease, including tick-borne infections [8]. Many wild animals, such as wild boars, hedgehogs, lizards, and snakes, have been identified as tick hosts [5, 9]. However, studies on lizard-tick associations are rare, especially in China. To date, only five tick species have been reported from lizards in China: *Amblyomma javanense*, *Amblyomma varanense, Amblyomma crassipes, Ixodes nipponensis*, and *Haenaphysalis sulcata* [5, 10]. Among them, only the immature stage (larva or nymph) of *H. sulcata* has been recorded on desert lizards in Xinjiang [11]. Meanwhile, lizards serve as a suitable host for many tick species [9, 10] and commonly share their habitats with domestic animals and human beings. A recent increase in human and animal infections from tick bites has been partly caused by a change in the hosts of ticks. Because two or three hosts are involved in the life history of many tick species, tick-associated agents, including bacteria, viruses, protozoa, and helminths, carried by lizards might infect domestic animals and eventually result in human infection [12, 13]. Different tick species are suited to carrying different pathogens. Thus, it is imperative to identify tick species rapidly and accurately.

Traditionally, ticks are identified using morphological methods and criteria [14]. However, these methods are insufficient for the identification of damaged, engorged, or immature specimens because of the loss or lack of morphological characteristics [15, 16]. Because of these limitations, only a limited number of experts such as taxonomists and trained technicians can identify ticks to species accurately [17]. Molecular taxonomy is an alternative method [18]. Molecular analyses based on DNA sequences could reveal species groups and assign unknown individuals to species. The PCR amplification of molecular markers has been developed and has become an essential method in the species identification of ticks [19–21]. Several genetic markers, such as the mitochondrial 12S ribosomal RNA gene (*12S rRNA*) [22, 23], 16S ribosomal RNA gene (*16S rRNA*) [24, 25], cytochrome c oxidase subunit 1 (*COI*) [23, 26], and the second internal transcribed spacer (*ITS2*) [27, 28], have been widely used for systematic studies on ticks.

In the present study, we amplified mitochondrial *12S rRNA*, *16S rRNA*, and *COI* genes to identify ticks isolated from desert lizards in Northwest China. The obtained sequences were used for alignment in conjunction with some tick sequences retrieved from GenBank. Our work is a first attempt to investigate ticks infesting lizards in the arid desert regions of Xinjiang, China, by both morphological examination and DNA sequencing. We further conducted a preliminary survey of sequence diversity at the intraspecies level to examine geographical and/or host specificity.

## Methods

### Study area and sampling procedures

**Fig. 1:**
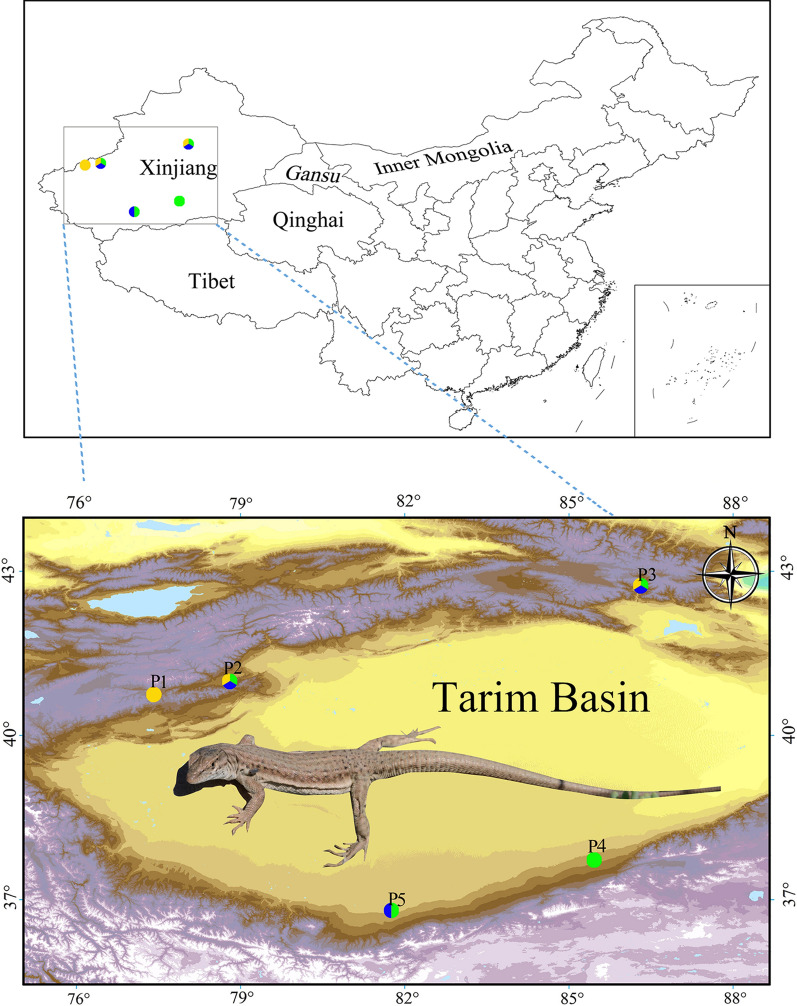
Sampling sites of hosts in the Tarim Basin and adjacent Yanqi Basin of Xinjiang. The site numbers P1–P5 correspond to those in Table 1. The species of ticks collected from each site are shown with different colors (yellow: *Haemaphysalis sulcata*, green: *Hy. asiaticum*, blue: *R. turanicus*). A general view of the multi-ocellated racerunner (*Eremias multiocellata*) is shown, with a tick near its armpit (photo by Jinlong Liu)

Thirty ticks were collected with tweezers from the body surfaces of lizards, which were identified as the multi-ocellated racerunner, *Eremias multiocellata*, via morphological determination following the commonly used classification system of lizards in China [29]. The lizards were hand-captured alive at three sites in the arid desert regions of the Xinjiang Uygur Autonomous Region of China (Fig. 1; Table 1). These sites are characteristic of arid deserts in the Tarim Basin and adjacent Yanqi Basin. In addition, 1 tick from brushwood (P4) and 47 ticks from 1 hedgehog (*Hemiechinus auritus*) (P5) were collected and used as reference samples, as shown in Additional file 1: Table S1. These ticks were collected and preserved separately in 2-ml sample tubes containing 2 ml 95% ethanol for later identification and DNA extraction. All 77 ticks were tentatively identified morphologically using microscopic examination and stored at the Chengdu Institute of Biology, Chinese Academy of Sciences. The protocol was approved by the medical ethics committee of Sichuan University (no. K2018056) and carried out under the National Guidelines for Experimental Animal Welfare (MOST of the People’s Republic of China, 2006).

**Table 1:** List of sampling localities and host species in this study

Site label	Locality	Hosts (individual number)
P1	Aheqi County, Kizilsu Kirghiz Autonomous Prefecture, Xinjiang	*E. multiocellata* (3)
P2	Aheqi County, Kizilsu Kirghiz Autonomous Prefecture, Xinjiang	*E. multiocellata* (1)
P3	Hejing County, Bayingol Mongolian Autonomous Prefecture, Xinjiang	*E. multiocellata* (2)
P4	Qiemo County, Bayingol Mongolian Autonomous Prefecture, Xinjiang	Free, not applicable
P5	Yutian County, Hotan Prefecture, Xinjiang	Hedgehog (1)

### DNA extraction, amplification, cloning, and sequencing protocols

The ticks were washed three times with phosphate-buffered saline (pH 7.4). Then, half of the bodies of the large ticks (width 3–8 mm) was cut into small pieces with a pair of sterile scissors. Total genomic DNA for each tick was extracted from the pieces of the half bodies of the large ticks or the whole bodies of the small ticks (width 1–2 mm), using the commercial TIANamp Genomic DNA Kit (TIANGEN Bio, Beijing, China) according to the manufacturer’s protocols. The extracted DNA samples were stored at −20 °C for further use. PCR primers specific for the ticks were synthesized by Tsingke Biological Technology Co., Ltd. (Chengdu, China). They were used to amplify *12S rRNA* [22], *16S rRNA* [30], and *COI* [23] gene fragments for each tick sample using the genomic DNA as template and PrimeSTAR Max DNA polymerase (TaKaRa Bio, Shiga, Japan), according to the manufacturer’s instructions. The following cyclic conditions were used: denaturation at 98 ℃ for 10 s; 15 s for annealing at a specific temperature; 1 min 10 s for elongation at 72 ℃. These three steps were repeated for 34 cycles. The negative control was treated with no template DNA and was included in all amplification runs. Successful PCR products were determined by electrophoresis on 1.5% agarose gel and were purified using a Universal DNA Purification Kit (TIANGEN Bio). The expected product sizes (excluding primer sequences) were approximately 320 bp of *12S rRNA*, 455 bp of *16S rRNA*, and 760 bp of *COI*. The PCR products were purified by excision of the band from the agarose gel using the Universal DNA Purification Kit (TIANGEN Bio) and were sequenced at Tsingke Biological Technology Co., Ltd.

### Sequence alignment and similarity analysis

Geneious Prime 2019.1.3 was used to study and edit the chromatograms. First, the obtained sequence data were preliminarily identified by GenBank searches using BLASTn (https://blast.ncbi.nlm.nih.gov/Blast.cgi). All the nucleotide sequences obtained in this study have been submitted to the GenBank database (Additional file 1: Table S1, Additional file 2: Table S2). Subsequently, all the sequences of each gene locus were multiple-aligned with a set of tick sequences for that locus. These sequences (34 *12S rRNA*, 78 *16S rRNA*, 72 *COI*) were retrieved from GenBank (Additional file 3: Tables S3) and aligned using the default options of Clustal W in MEGA v.7.0.26 [31] and refined manually. Finally, the uncorrected distances of *12S rRNA*, *16S rRNA*, and *COI* sequences were calculated with MEGA within and between each species in the same genus, respectively.

### Network reconstruction

To present the relationships among haplotypes within species and their relatedness with the geographical origin or hosts, the median-joining (MJ) network reconstruction method [32] was applied to *Haemaphysalis sulcata*, *Rhipicephalus turanicus*, and *Hyalomma asiaticum* sequences. First, distinct sequence types (haplotypes) were defined by using the program DAMBE v.5.2.30 [33]; these were used for the subsequent analyses. The MJ network method followed by the maximum parsimony (MP) option to clean up the network was implemented using the program NETWORK v5.0.0.3 (available at http://www.fluxus-engineering.com/sharenet.htm). Meanwhile, to further assess the possibility of shared ancestry for each tick species, we did haplotype network analyses with other sequence types from other regions or hosts samples. Eight representative *16S rRNA* sequences of *H. sulcata*, nine representative *COI* sequences of *H. sulcata*, 81 representative *COI* sequences of *R. turanicus*, and 104 representative *COI* sequences of *Hy. asiaticum* were retrieved from GenBank for comparisons, respectively (Additional file 5: Table S5, Additional file 6: Table S6, Additional file 7: Table S7, Additional file 8: Table S8).

## Results

### Tick sample collection from lizards

In total, 30 ticks parasitizing lizards (identified as *E. multiocellata*) were collected from three sampling sites in Xinjiang. Six lizards were captured alive by hand. The total prevalence of ticks infested was 50% (Table 2). The majority of ticks were found in the lizards’ armpits and crotch areas, and a few ticks were found on the head, on the sides of the chest, or in the pericloacal region. Based on morphological keys and descriptions in previous reports [11], preliminary examination identified these ticks to three species of three genera: *Hy. asiaticum*, *R. turanicus*, and *H. sulcata*.

**Table 2:** List of sampling localities, lizard host species, tick species, and sample size in this study

Site label	Hosts (infested/total)	Ticks (total)		*H. sulcata*		*Hy. asiaticum*		*R. turanicus*	
		No. %		No. %		No. %		No. %	
P1	*E. multiocellata* (1/3)	2	6.67	2	6.67	0	0	0	0
P2	*E. multiocellata* (1/1)	12	40	4	13.33	6	20	2	6.66
P3	*E. multiocellata* (1/2)	16	53.33	11	36.67	4	13.33	1	3.33
Total	3/6	30	100	17	56.67	10	33.33	3	13.33

P1, P2, and P3 correspond to those in Table 1

### Sequence characteristics

The PCR amplification of each locus resulted in amplicons of the expected lengths (approximately 320 bp for *12S rRNA,* 455 bp for *16S rRNA*, and 760 bp for *COI*). The estimated mean frequencies of the GC were as follows: 23.5% for *12S rRNA*, 21.31% for *16S rRNA*, and 32.7% for *COI.* Thus, both the lengths and GC content were within the range of tick species [21]. Overall, sequence searching with BLASTn (megablast) revealed over 90% identity with *Hy. asiaticum*, *R. turanicus*, or *H. sulcata*. For *12S rRNA*, the similarities between sequences in this study and reference sequences from GenBank varied from 94.7 to 96% for *H. sulcata*, 99.5% to 100% for *Hy. asiticum*, and 99.6% to 100% for *R. turancus*. For *COI*, the similarities between sequences in this study and reference sequences from GenBank varied from 90% to 91.1% for *H. sulcata*, 99.5% to 100% for *Hy. asiticum*, and 100% for *R. turancus*. For *16S rRNA*, the similarities between sequences in this study and reference sequences from GenBank varied from 98.7 to 100% for *H. sulcata*, 99.7% for *Hy. asiticum*, and 100% for *R. turancus*.

For *12S rRNA*, the similarities of intraspecific sequences of *H. sulcata* varied from 94.3 to 100%, while similarities of interspecific sequences within genera were estimated from 85.8 to 90.5% (Table 3). The similarities of intraspecific sequences of *Hy. asiaticum* varied from 99.6 to 100%, while similarities of interspecific sequences within genera were estimated from 86 to 92.6%. The similarities of intraspecific sequences of *R. turancus* varied from 99.2 to 99.6%, while similarities of interspecific sequences within genera were estimated from 85.4 to 95.2% .

**Table 3:** Average value of uncorrected pairwise distance and the range of similarities between sequences of ticks from the same or from difference species

		Gene	*H. sulcata*	*Hy. asiticum*	*R. turancus*
Similarities within tick species (%)	Between sequences in this study and reference sequences from GenBank	*12S rRNA*	94.7−96	99.5−100	99.6-100
		*COI*	90−91.1	99.5−100	100
		*16S rRNA*	98.7−100	99.7	100
Similarities between sequences (%)	Within species	*12S rRNA*	94.3−100	99.6−100	99.2−99.6
		*COI*	90.5−100	98.9−100	93.3−100
		*16S rRNA*	98.7−100	95.3−99.7	98.3−100
	Between species	*12S rRNA*	85.8−90.5	86−92.6	85.4−95.2
		*COI*	82.9−87.7	84.2−92.4	84.5−91.5
		*16S rRNA*	79.1−91.8	85−92.3	83.5−94.2
Mean value of uncorrected genetic distance between sequences	Within species	*12S rRNA*	0.012	0	0.005
		*COI*	0.022	0.002	0.019
		*16S rRNA*	0.009	0.02	0.002
	Between species	*12S rRNA*	0.12	0.109	0.096
		*COI*	0.148	0.117	0.131
		*16S rRNA*	0.124	0.077	0.112

For *COI*, the similarities of intraspecific sequences of *H. sulcata* varied from 90.5 to 100%, while similarities of interspecific sequences within genera were estimated from 82.9% to 87.7% (Table 3). The similarities of intraspecific sequences of *Hy. asiaticum* varied from 98.9 to 100%, while similarities of interspecific sequences within genera were estimated from 84.2 to 92.4%. The similarities of intraspecific sequences of *R. turancus* varied from 93.3 to 100%, while similarities of interspecific sequences within genera were estimated from 84.5 to 91.5%.

For *16S rRNA*, the similarities of intraspecific sequences of *H. sulcata* varied from 98.7 to 100%, while similarities of interspecific sequences within genera were estimated from 79.1 to 91.8% (Table 3). The similarities of intraspecific sequences of *Hy. asiticum* varied from 95.3 to 99.7%, while similarities of interspecific sequences within genera were estimated from 85 to 92.3%. The similarities of intraspecific sequences of *R. turancus* varied from 98.3 to 100%, while similarities of interspecific sequences within genera were estimated from 83.5 to 94.2%.

In addition, the tick species identified in this study were most closely related to the same species, but exhibited higher differences from other species in the same genus (Table 3).

### Median-joining network

**Fig. 2:**
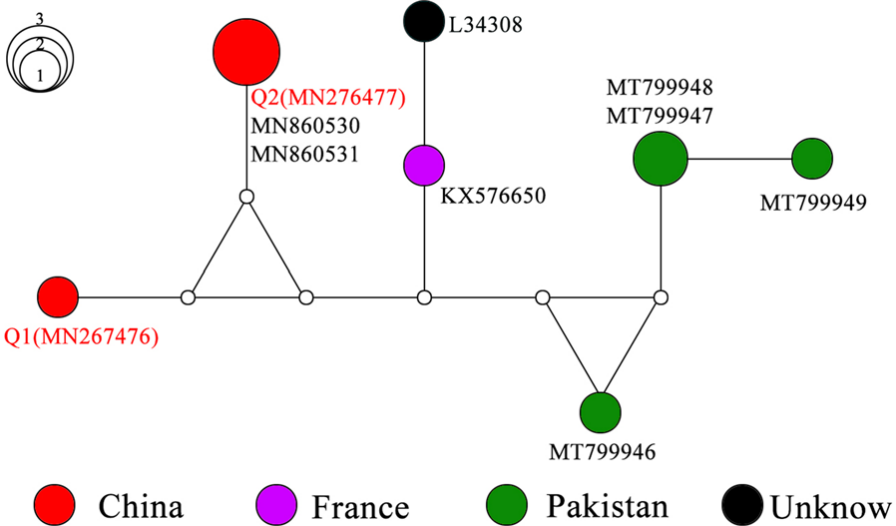
Median-joining network based on the *16S rRNA* haplotype of *Haemaphysalis sulcata*. Solid circles of the network indicate the haplotypes, and small hollow circles indicate median vectors inferred by NETWORK software. Different filled patterns represent the corresponding geographical origin from which the haplotype was sampled. The size of the circles roughly represents the numbers of sequences carrying the haplotype, with the scale given beside the network. The representative reference sequences retrieved from GenBank are listed in Additional file 5: Table S5

**Fig. 3:**
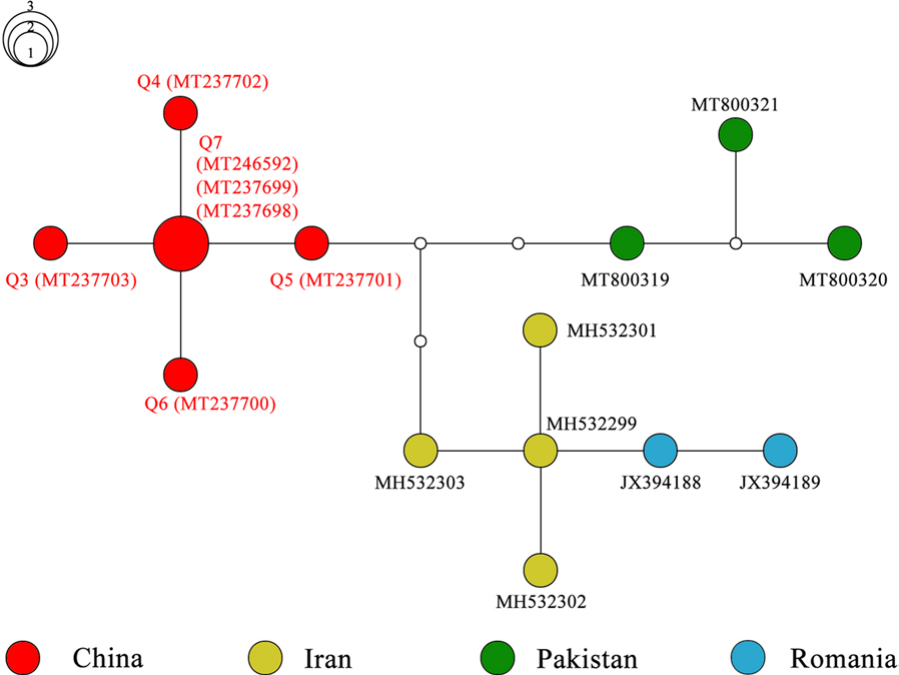
Median-joining network based on *COI* haplotype of *Haemaphysalis sulcata*. Solid circles of the network indicate the haplotypes, and small hollow circles indicate median vectors inferred by the NETWORK software. Different filled patterns represent the corresponding geographical origin from which the haplotype was sampled. The size of the circles roughly represents the numbers of sequences carrying the haplotype, with the scale given beside the network. The representative reference sequences retrieved from GenBank are listed in Additional file 6: Table S6

To further evaluate the relationships among the intraspecific genes of lizard ticks, MJ networks were constructed using the MJ algorithm network method. The network of seven haplotypes of *16S rRNA* (alignment length of 266 bp) for *H. sulcata* is shown as Fig. 2. Q1 and Q2, collected from lizards in Northwest China, were more closely related to each other than to the other sequences from different countries. Meanwhile, the network of 14 haplotypes of *COI* (alignment length of 550 bp) for *H. sulcata* is shown as Fig. 3. The main feature of the haplotype distribution was the occurrence of a clear geographical structuring. Apparently, the *COI* haplotype network of *H. sulcata* from the lizards is centered around haplotype Q7. Furthermore, compared with those from Iran, Pakistan, and Romania, the *H. sulcata* from China did cluster together.

**Fig. 4:**
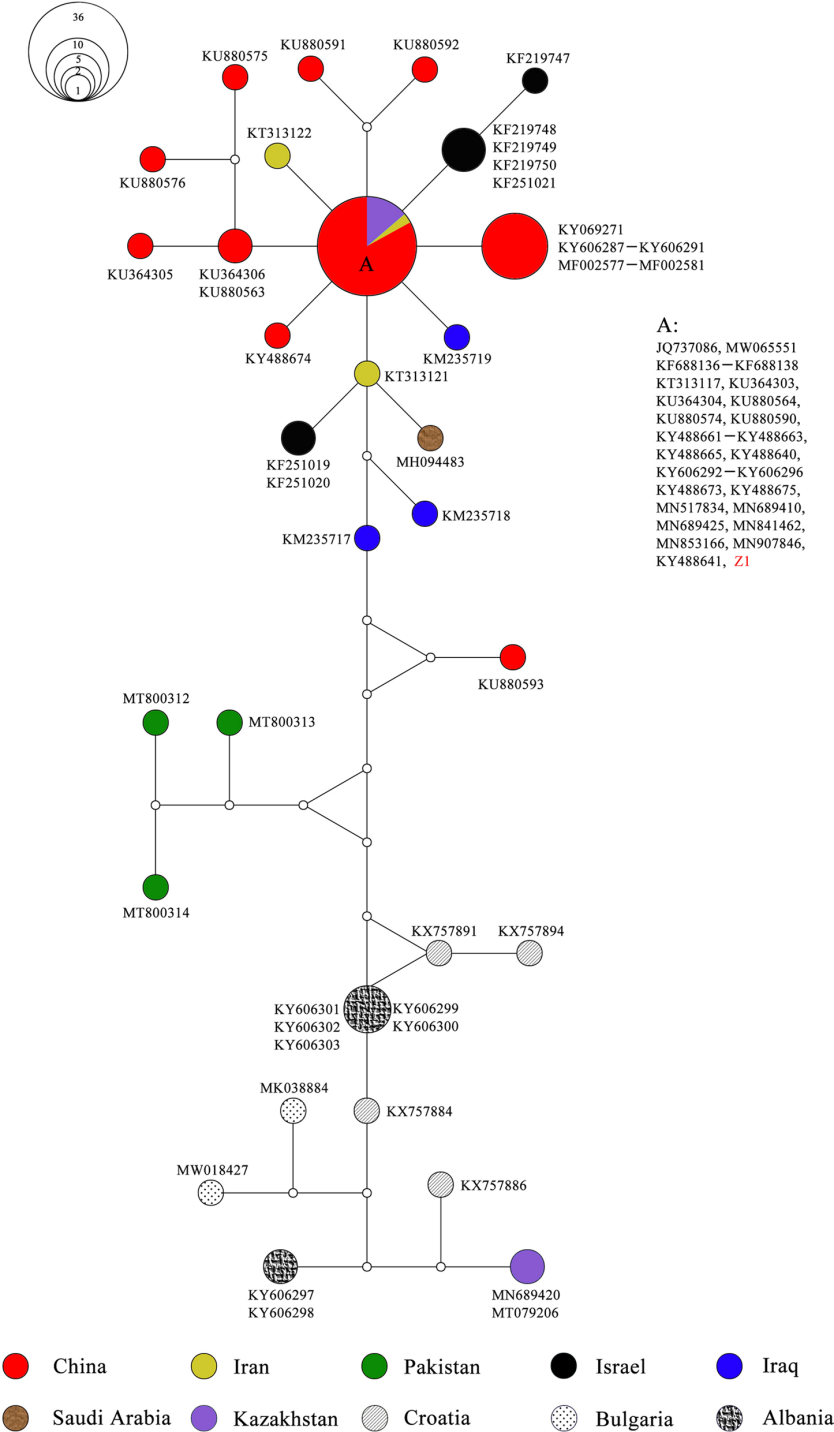
Median-joining network based on *CO1* haplotypes of *Rhipicephalus turanicus*. Solid circles of the network indicate the haplotypes, and small hollow circles indicate median vectors inferred by NETWORK software. The size of the solid circles roughly represents the numbers of sequences carrying the haplotype, with the scale given beside the network. Different filled patterns represent the corresponding geographical origin from which the haplotype was sampled. The representative reference sequences retrieved from GenBank are listed in Additional file 7: Table S7

To further assess the possibility of shared ancestry for *R. turanicus*, we conducted haplotype network analysis with other sequence types from other regions/hosts. Eighty-one representative *COI* sequences of *R. turanicus* were retrieved from GenBank for comparison (see Additional file 7: Table S7). The network based on 31 *COI* haplotypes (496 bp) of *R. turanicus* is shown in Fig. 4. It exhibited no apparent geographic structure rather than a complex pattern, with the network comprising multiple configurations, including one star-like network. The *R. turanicus* from China did not cluster together; they were instead paraphyletic with those from other countries including Iran, Kazakhstan, Israel, and Iraq. It also showed that most haplotypes of *R. turanicus* from China were closely related to each other with the exception of one sequence (KU880593). Interestingly, Z1 (6 sequences obtained in this study) and the other 24 sequences from China shared the same haplotype (A) as 1 sequence from Iran (KT313117) and 5 from Kazakhstan (MN689425, MN689410, MN841462, MN907846, and MN853166). Overall, at the intraspecific level, the haplotype network could directly reflect the small genetic distances between the Z1 haplotype obtained in this study and other adjacent haplotypes.

**Fig. 5:**
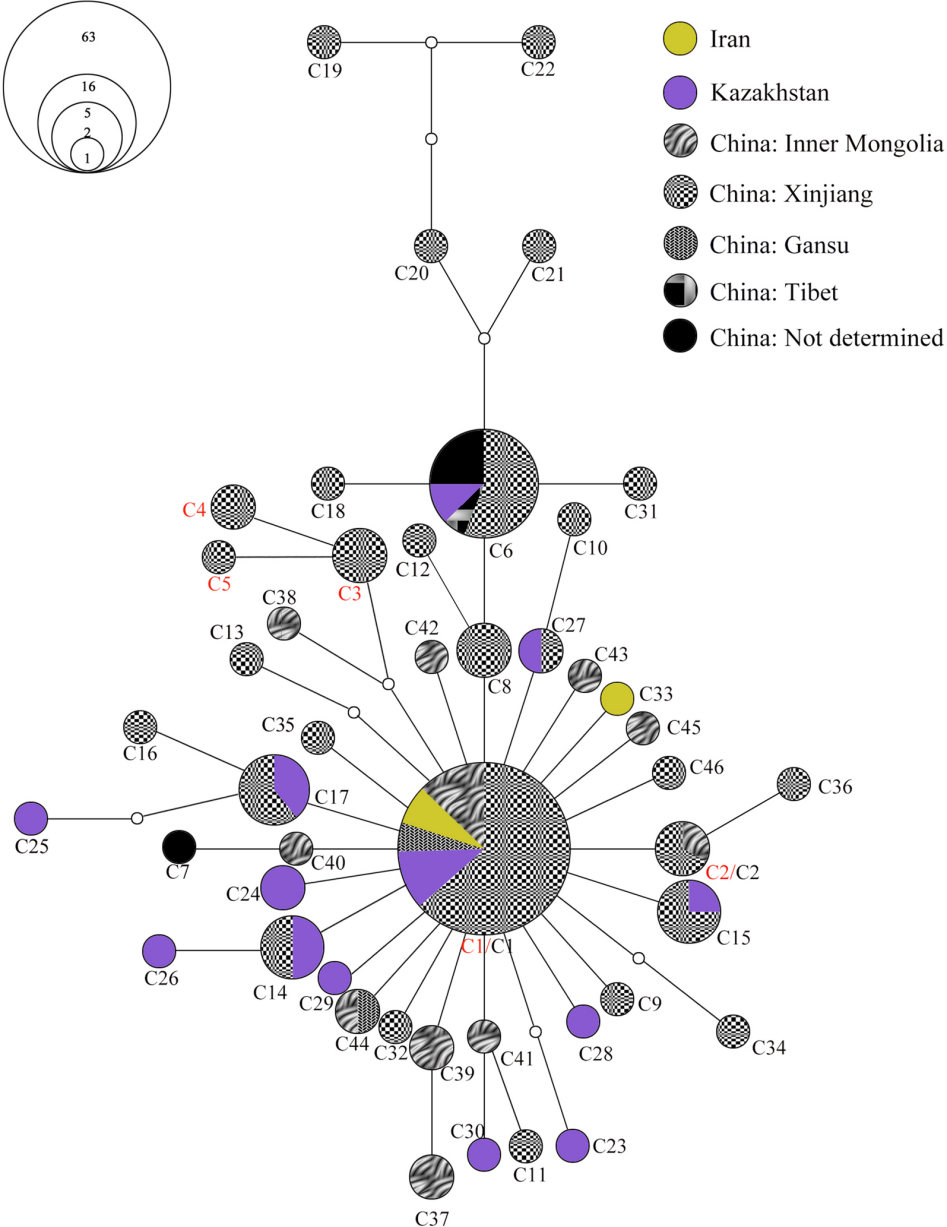
Median-joining network based on *CO1* haplotype of *Hyalomma asiaticum*. Solid circles of the networks indicate the haplotypes, and small hollow circles indicate median vectors inferred by NETWORK software. The size of the solid circles roughly represents the numbers of sequences carrying the haplotype, with the scale given beside the network. Different filled patterns represent the corresponding geographical origin from which the haplotype was sampled. The representative reference sequences retrieved from GenBank are listed in Additional file 8: Table S8

In addition, to further evaluate the possibility of shared ancestry for *Hy. asiaticum*, we did haplotype network analysis with other sequence types from other regions/hosts. One hundred four representative *COI* sequences of *Hy. asiaticum* were retrieved from GenBank for comparison (see Additional file 8: Table S8). For *Hy. asiaticum*, the MJ network produced a complex pattern (Fig. 5), with the network comprising multiple configurations, including one star-like network, with the central sequences being the most frequent haplotype (C1). Although 46 haplotypes were detected, the network shows relatively shallow genetic divergence and little evidence of an overt geographic structure. Moreover, the haplotype C1 was shared by 63 sequences, and among them, 4 were from Iran (KP219869, KP219864, KP219860, and KP208951), 7 from Kazakhstan (MN892553, MN961479, MN907845, KU364334, KU364332, KU364324, and KU364317), and the rest from China (including Inner Mongolia, Xinjiang, Gansu, etc.). Two sequences (MT237666 and MT237693), obtained from hedgehog in Xinjiang, shared the same C2 haplotype with one sequence from sheep in Inner Mongolia, China. In addition, the *COI* haplotype network of *Hy. asiaticum* could intuitively reflect the distances between the five obtained haplotypes in this study.

## Discussion

This study is the first to investigate ticks on lizards from the arid desert regions of Xinjiang, China, using both morphological examination and molecular techniques. The results show that the 30 ticks from *E. multiocellata* lizards at 3 sites in this study belong to 3 genera and 3 species (*H. sulcata*, *Hy. asiaticum*, and *R. turanicus*). Among them, *H. sulcata* was the most abundant species and *R. turanicus* the rarest, with only four ticks*. Hy. asiaticum* and *R. turanicus* were reported in lizards in Northwest China for the first time.

The tick *H. sulcata* is one of the three host tick species. The adults have been reported to usually feed on domestic and wild ungulates, while the immature stages have a special preference for reptiles [5, 11, 34]. In Turkey, *H. sulcata* was once reported on *Apathya cappadocica*, *Stellagama stellio*, and *Ophisops elegans* [35]. Recently, six lizard species in Turkey have been found to be reptilian hosts of *H. sulcata* [36]*.* Consistent with previous researches in China [5, 11], we found immature *H. sulcata* feeding on lizards. In this study, the *H. sulcata* haplotypes obtained from the lizards have a close relationship with those derived from GenBank. It is notable that one of the *16S rRNA* sequences (MN276477), collected from the lizard, and the two sequences of *H. sulcata* (MN860530, MN860531) were found to be identical. The three shared the same Q2 haplotype (Fig. 2). In addition, for *COI*, one sequence with accession MT237701, collected from the lizard, is three substitutions away from those from Iran (MH532303) and Pakistan (MT800319) (Fig. 3). Therefore, we can conservatively infer that 17 of the ticks collected from the lizards can be identified to the species *H. sulcata* with confidence.

Interestingly, we found not only *H. sulcata* feeding on the lizards, but also found two local tick species that are dominant in Xinjiang, *Hy. asiaticum* and *R. turanicus.* A previous survey of ticks on livestock also reported that *H. sulcata*, *Hy. asiaticum*, and *R. turanicus* were found in the Tarim Basin in Xinjiang. As the prevalent species in Xinjiang, *Hy. asiaticum* has a broad host range, reaching more than 50 species, mainly artiodactyls and smaller mammals [37, 38].

*Hyalomma asiaticum* is also a three-host tick species. All hosts can be opportunistically infested at all stages of *Hy. asiaticum* [39]. The chief hosts of its immature stages are smaller mammals (e.g., rodents, hedgehogs, and shrews), while carnivores, birds, and reptiles are seemingly secondary or occasional hosts [40]. It was recorded that the immature stages of *Hy. asiaticum* were once collected and identified from the Przewalski’s wonder gecko *Teratoscincus przewalskii* [39], albeit without indicating its geographical origin. This is the first report on the immature stages of infestation of *Hy. asiaticum* on racerunner lizards in Xinjiang, Northwest China.

*Hyalomma asiaticum* is widely distributed in Asia, from Syria in the West to eastern China in the East [39], exhibiting great geographical and individual variability. In contrast to the population structure of *Hy. asiaticum* revealed by mitochondrial and nuclear genome [41], our data did not recover the geographical differentiation between Xinjiang and Inner Mongolia (Fig. 5), even between China and Kazakhstan and Iran. This inconsistency may be explained by the incomplete lineage sorting due to limited polymorphic sites in the *COI* gene segments. No apparent geographical structure is observed that links the haplotypes with host taxa (Additional file 8: Table S8). In an exponentially growing population starting from a small source group, a "star phylogeny" can be anticipated with many genealogical lineages tracing to a restricted span of times near the initial population expansion [42]. As shown in Fig. 5, a common ancestral-like haplotype (C1) lies at the star’s center, and recent derivatives are connected to it independently by short branches. This star-like network suggests that *Hy. asiaticum* might have experienced recent expansion. Furthermore, the pattern of sequence diversity within *Hy. asiaticum* provides evidence for widespread tick infestations across host taxa and across geographical locations, ranging from different provinces in China to adjacent Kazakhstan, and even further in Iran. In other words, it seems that there is no spatially scale-dependent host specificity for *Hy. asiaticum*. This pattern of low host specificity of *Hy. asiaticum* is similar to that observed in some other ticks. For example, a recent study revealed that there is limited host specificity and no clear relation to the geographical distribution of *Amblyomma triste* and *A. tigrinum* immature stages from Argentina [43].The cause of the low geographical and host specificity in *Hy. asiaticum* is unknown, but we speculate it may stem from its generalist behavior [39]. However, there are still unique hedgehog-tick haplotypes (C3, C4 and C5) in Xinjiang, China*.* It is of vital importance to study the specificity of ticks distinguishing their life stage [43, 44]. After all, a generalist behavior may result in dissimilar levels of infestation across a range of usual hosts [43].

In previous studies, *R. turanicus* was recognized as a widespread species in desert and semi-desert areas in the southern region of Xinjiang [45, 46]. However, the abundance of this species seems to be partly reduced owing to the limited sample size in the present study. Although this species has been reported to feed on lizards [47], the record of *R. turanicus* collected from lizards in China was blank until we found the three lizard ticks in this study. As shown in Fig. 4, in the *R. turanicus* clade, one haplotype (Z1, three sequences from lizards and three from hedgehog) shared an identical haplotype (A) with 24 Chinese *R. turanicus* as well as some sequences from Kazakhstan and Iran derived from GenBank. A star-like network surrounding the common ancestral-like haplotype (A) suggests that *R. turanicus* might have experienced recent population expansion. To enrich the knowledge of tick species infesting lizards and their potential to cause tick-borne diseases, more investigations are needed. Reptile ticks could perhaps be believed to be less widely distributed in China only because of the lack of investigation on them.

*Eremias multiocellata* is highly adapted to desert and semi-desert habitats, with a wide distribution from northern China, across Mongolia to the Tuva Republic of Russia [29, 48]. Several factors may affect the distribution of tick species, such as the climate, human land-use patterns, geographical habitats, and hosts [9, 10]. The Tarim Basin hosts the largest shifting-sand desert in central Asia, the Taklimakan Desert. As an endorheic basin in southern Xinjiang, the basin is surrounded by the Tien Shan to the north, the Pamirs to the west, the Kunlun Mountains to the south, and the Altun Mountains to the east. Overall, the climate in the basin is extremely dry as the mountains have blocked out moist air from the sea since the beginning of the Pliocene [49]. Various landscapes in the Tarim Basin are composed of desertified grassland, salinized desert, and human- and animal-inhabited oases [6, 45]. The Yanqi Basin is located at the southeastern Tien Shan and separated from the Tarim Basin by the eastward extension of the Tien Shan (i.e. Kuruktagh), whose terrain slopes up from the northwest down to the southeast. The northwest is mountainous, and the south is low-lying desert [50]. These together were consistent with the adapted living environments of the three tick species found in the lizards [38, 46]. Accordingly, we suggest that the characteristics found in this study of ticks in Xinjiang are closely related to the geographical environments. Further study is necessary to test whether climatic condition, lizard ecological traits, and host phylogeny explain tick prevalence across desert lizard species in an explicit geographical framework.

Xinjiang, adjacent to eight countries (Russia, Kazakhstan, Kyrgyzstan, Tajikistan, Pakistan, Mongolia, India, and Afghanistan), is an important transportation hub. The economic system of Xinjiang is mainly agriculture and animal husbandry; thus, farmers and herders are in close contact with livestock, reptiles, and ticks. Therefore, the outlook for the spread and epidemic risk of tick-borne disease in this area is grim, and the potential for the early warning, prevention, and control of tick-borne diseases should be improved. Further studies on the storage and transmission of pathogens in ticks are needed to help us control ticks and tick-borne diseases efficiently.

## Conclusions

Based on both morphological and molecular techniques, ticks parasitic on lizards have been identified for the first time in the arid desert regions of Xinjiang in China. The detected ticks belong to three species and three genera, including *Hy. asiaticum*, *R. turanicus*, and *H. sulcata*. *Eremias multiocellata* is an atypical lizard host of the three tick species. Notably, two species of ticks, *Hy. asiaticum* and *R. turanicus*, have been collected and identified from lizards in China for the first time to our knowledge. Star-like networks for *R. turanicus* and *Hy. asiaticum* suggest both of them might have experienced recent population expansion. The characteristics of ticks found in our study in Xinjiang are closely related to the geographical environments. Our findings could enrich our knowledge about tick species infesting wild animals and help us to understand the association between lizards and ticks in Xinjiang.

## Supplementary Information

**Additional file 1: Table S1.** List of the voucher number, origin, haplotype number, and GenBank accession numbers of the ticks obtained from hedgehog and brushwood in this study.

**Additional file 2: Table S2.** List of the voucher number, origin, stage, haplotype number, and GenBank accession numbers of the ticks obtained from lizards in this study.

**Additional file 3: Table S3. **List of the tick species retrieved from GenBank for sequence similarity analysis, with accession numbers and references.

**Additional file 4: Table S4.** List of the host, origin, tick species, haplotype number, and GenBank accession numbers of the ticks obtained from this study and used for median-joining network presented in Figs. 2, 3, 4, 5.

**Additional file 5: Table S5.** Accession numbers for eight *16S rRNA* gene sequences of *Haemaphysalis* *sulcata* downloaded from GenBank and used for the median-joining network presented in Fig. 2.

**Additional file 6: Table S6. **Accession numbers for 9 *COI* gene sequences of *Haemaphysalis* *sulcata* downloaded from GenBank and used for the median-joining network presented in Fig. 3.

**Additional file 7: Table S7. **Accession numbers for 81 *COI* gene sequences of *Rhipicephalus turanicus *downloaded from GenBank and used for the median-joining network presented in Fig. 4.

**Additional file 8: Table S8.** Accession numbers for 104 *COI* gene sequences of* Hyalomma asiaticum* downloaded from GenBank and used for the median-joining network presented in Fig. 5.

## Data Availability

All data generated or analysed during this study are included in this published article [and its additional files].

## Abbreviations

*Hy. asiaticum*: *Hyalomma asiaticum*
*H. sulcata*: *Haemaphysalis sulcata*
*R. turanicus*: *Rhipicephalus turanicus*
*12S rRNA*: 12S ribosomal RNA gene
*16S rRNA*: 16S ribosomal RNA gene
*COI*: Cytochrome c oxidase subunit 1
PCR: Polymerase chain reaction
MJ: Median-joining
MP: Maximum parsimony
