# Supplementary material for: Records of three mammal tick species parasitizing an atypical host, the multi-ocellated racerunner lizard, in arid regions of Xinjiang, China
Source: Parasit Vectors. 2021 Mar 4;14:135. doi: 10.1186/s13071-021-04639-z (PMC7931338; doi:10.1186/s13071-021-04639-z)
Supplement: Supplementary file 1 — Additional file 1: Table S1. List of the voucher number, origin, haplotype number, and GenBank accession numbers of the ticks obtained from hedgehog and brushwood in this study. [file 13071_2021_4639_MOESM1_ESM.docx]

Table S1. List of the voucher number, origin, haplotype number and GenBank accession numbers of the ticks obtained from hedgehog and brushwood in this study.

| Voucher number | Haplotype number | Origin | GenBank accession | | |
| --- | --- | --- | --- | --- | --- |
|  |  |  | *12S rRNA* | *16S rRNA* | *CO1* |
| R1 | H22 | P4 | MN267435 | / | MT237692 |
| R2 | H22 | P5 | MN267450 | / | MT237669 |
| R3 | H22 | P5 | MN267451 | / | MT237679 |
| R4 | H22 | P5 | MN267454 | / | MT237682 |
| R5 | H22 | P5 | MN267464 | / | MT237671 |
| R6 | H22 | P5 | MN267467 | / | MT237686 |
| R7 | H22 | P5 | MN267460 | / | MT237694 |
| R8 | H15 | P5 | / | / | MT237655 |
| R9 | H15 | P5 | / | / | MT237673 |
| R10 | H15 | P5 | / | / | MT237660 |
| R11 | H15 | P5 | / | / | MT237674 |
| R12 | H15 | P5 | / | / | MT237670 |
| R13 | H15 | P5 | / | / | MT237657 |
| R14 | H15 | P5 | / | / | MT237683 |
| R15 | H15 | P5 | / | / | MT237665 |
| R16 | H15 | P5 | / | / | MT237664 |
| R17 | H15 | P5 | / | / | MT237663 |
| R18 | H23 | P5 | / | / | MT237687 |
| R19 | H24 | P5 | MN267448 | / | MT237689 |
| R20 | H25 | P5 | MN267449 | / | MT237668 |
| R21 | H26 | P5 | / | / | MT237659 |
| R22 | H27 | P5 | MN267452 | / | MT237677 |
| R23 | H28 | P5 | MN267453 | / | MT237693 |
| R24 | H29 | P5 | / | MN267478 | MT237680 |
| R25 | H30 | P5 | MN267456 | / | MT237690 |
| R26 | H31 | P5 | MN267455 | / | / |
| R27 | H32 | P5 | / | MN267479 | MT237706 |
| R28 | H33 | P5 | / | MN267480 | / |
| R29 | H34 | P5 | / | MN267481 | / |
| R30 | H35 | P5 | / | MN267482 | MT237707 |
| R31 | H36 | P5 | MN267457 | / | / |
| R32 | H37 | P5 | MN267458 | / | / |
| R33 | H38 | P5 | MN267459 | / | MT237666 |
| R34 | H39 | P5 | MN267466 | / | MT237696 |
| R35 | H40 | P5 | MN267469 | / | MT237691 |
| R36 | H40 | P5 | MN267463 | / | MT237658 |
| R37 | H40 | P5 | MN267465 | / | MT237685 |
| R38 | H41 | P5 | MN267470 | / | / |
| R39 | H42 | P5 | / | / | MT237688 |
| R40 | H43 | P5 | / | MN267483 | / |
| R41 | H10 | P5 | / | / | MT237708 |
| R42 | H44 | P5 | MN267468 | / | MT240263 |
| R43 | H45 | P5 | / | / | MT240264 |
| R44 | H46 | P5 | MN267461 | / | MT237656 |
| R45 | H47 | P5 | / | MN267484 | / |
| R46 | H37 | P5 | MN267462 | / | / |
| R47 | H40 | P5 | MN267471 | / | MT237684 |
| R48 | H40 | P5 | MN267472 | / | / |

P4 and P5 correspond those in Table 1.
