# Supplementary material for: Records of three mammal tick species parasitizing an atypical host, the multi-ocellated racerunner lizard, in arid regions of Xinjiang, China
Source: Parasit Vectors. 2021 Mar 4;14:135. doi: 10.1186/s13071-021-04639-z (PMC7931338; doi:10.1186/s13071-021-04639-z)
Supplement: Supplementary file 2 — Additional file 2: Table S2. List of the voucher number, origin, stage, haplotype number, and GenBank accession numbers of the ticks obtained from lizards in this study. [file 13071_2021_4639_MOESM2_ESM.docx]

Table S2. List of the voucher number, origin, stage, haplotype number and GenBank accession numbers of the ticks obtained from lizards in this study.

| Voucher number | Haplotype number | Stage | Origin | GenBank accession | | |
| --- | --- | --- | --- | --- | --- | --- |
|  |  |  |  | *12S rRNA* | *16S rRNA* | *COI* |
| L1 | H1 | Larva | P2 | MN267437 | / | MT237700 |
| L2 | H2 | Larva | P2 | MN267436 | / | / |
| L3 | H3 | Nymph | P3 | / | MN267476 | / |
| L4 | H4 | Larva | P3 | MN267441 | / | / |
| L5 | H4 | Nymph | P3 | MN267443 | / | / |
| L6 | H4 | larva | P3 | MN267439 | / | / |
| L7 | H5 | Nymph | P3 | MN267442 | / | MT237702 |
| L8 | H6 | Larva | P3 | MN267444 | / | / |
| L9 | H7 | Nymph | P3 | / | / | MT246592 |
| L10 | H8 | Nymph | P3 | MN267445 | / | / |
| L11 | H9 | Nymph | P3 | MN267438 | / | MT237698 |
| L12 | H10 | Larva | P1 | / | / | MT237709 |
| L13 | H10 | Larva | P3 | / | / | MT237704 |
| L14 | H11 | Larva | P3 | / | / | MT237697 |
| L15 | H12 | Larva | P3 | / | / | MT237662 |
| L16 | H13 | Nymph | P3 | / | / | MT237653 |
| L17 | H14 | Nymph | P3 | MN267440 | / | / |
| L18 | H15 | Larva | P3 | / | / | MT237676 |
| L19 | H15 | Nymph | P1 | / | / | MT237667 |
| L20 | H15 | Nymph | P3 | / | / | MT237661 |
| L21 | H15 | Larva | P1 | / | / | MT237675 |
| L22 | H15 | Larva | P1 | / | / | MT237672 |
| L23 | H15 | Nymph | P1 | / | / | MT237654 |
| L24 | H15 | Nymph | P1 | / | / | MT237695 |
| L25 | H16 | Nymph | P1 | / | MN267477 | MT237703 |
| L26 | H17 | Nymph | P1 | MN267447 | / | MT237699 |
| L27 | H18 | Larva | P1 | / | / | MT237701 |
| L28 | H19 | Nymph | P1 | / | / | MT237705 |
| L29 | H20 | Nymph | P1 | MN267446 | / | / |
| L30 | H21 | Nymph | P1 | / | / | MT237681 |

P1, P2, P3 correspond to those in Table 1.
