# Supplementary material for: Records of three mammal tick species parasitizing an atypical host, the multi-ocellated racerunner lizard, in arid regions of Xinjiang, China
Source: Parasit Vectors. 2021 Mar 4;14:135. doi: 10.1186/s13071-021-04639-z (PMC7931338; doi:10.1186/s13071-021-04639-z)
Supplement: Supplementary file 3 — Additional file 3: Table S3. List of the tick species retrieved from GenBank for sequence similarity analysis, with accession numbers and references. [file 13071_2021_4639_MOESM3_ESM.docx]

Table S3. List of the tick species retrieved from GenBank for sequence similarity analysis, with accession numbers and references

| Ticks species | Voucher number | GenBank accession | | | References |
| --- | --- | --- | --- | --- | --- |
|  |  | *12S rRNA* | *16S rRNA* | *COI* |  |
| *Haemaphysalis aborensis* | \ | \ | KC170735 | \ | [1] |
| *Haemaphysalis asiatica* | \ | \ | KC170734 | \ | [1] |
| *Haemaphysalis bancrofti* | HB3F | \ | \ | MN106723 | [2] |
| *Haemaphysalis bispinosa* | PK38 | \ | \ | MN106409 | \ |
| *Haemaphysalis campanulata* | NCCDC1672 | \ | MG696719 | \ | \ |
| *Haemaphysalis chordeilis* | PSOC | \ | \ | MN991269 | [3] |
| *Haemaphysalis concinna* | \ | \ | KU170523 | KU170516 | [4] |
| *Haemaphysalis cornigera* | \ | \ | AB819174 | \ | [5] |
| *Haemaphysalis cretica** | \ | U95870 | L34308 | \ | [6, 7] |
| *Haemaphysalis danieli* | \ | \ | MH394440 | \ |  |
| *Haemaphysalis doenitzi* | [1048531](https://www.ncbi.nlm.nih.gov/Taxonomy/Browser/wwwtax.cgi?id=1048531) | JQ346679 | JF979402 | JQ346688 | [8, 9] |
| *Haemaphysalis elliptica* | Shingwedzi2 | HM068955 | HM068961 | \ | \ |
| *Haemaphysalis erinacei* | ABL4 | \ | KU880550 | KU880615 | \ |
| *Haemaphysalis flava* | AJ201803B-hf1 | \ | MN650207 | MN650208 | \ |
| *Haemaphysalis formosensis* | Hfo-B | \ | AB819195 | \ | [5] |
| *Haemaphysalis humerosa* | \ | \ | \ | MN106725 | [2] |
| *Haemaphysalis hystricis* | JBB03_2 | \ | \ | MK573784 | [10] |
| *Haemaphysalis inermis* | i43 | \ | \ | MH532295 | \ |
| *Haemaphysalis intermedia* | 51 | \ | MH044718 | \ | \ |
| *Haemaphysalis juxtakochi* | juxtakochiN1002 | \ | MH513303 | MH513245 | [11] |
| *Haemaphysalis kitaokai* | \ | \ | MH208568 | \ | [12] |
| *Haemaphysalis kopetdaghica* | B-1115 | \ | MT302757 | MT308585 | \ |
| *Haemaphysalis lagostrophi* | E1LaF | \ | \ | MN686569 | \ |
| *Haemaphysalis lagrangei* | \ | \ | MG788690 | \ | [13] |
| *Haemaphysalis leachi* | MARK45 | \ | MN661151 | MN663156 | [3] |
| *Haemaphysalis lemuris* | \ | \ | \ | JX470178 | [14] |
| *Haemaphysalisleporispalustris* | \ | \ | \ | MN663154 | [3] |
| *Haemaphysalis longicornis* | CT-HL-484 | \ | \ | MT068544 | [15] |
| *Haemaphysalis mageshimaensis* | \ | \ | AB819213 | \ | [5] |
| *Haemaphysalis megaspinosa* | \ | \ | AB819216 | \ | [5] |
| *Haemaphysalis montgomeryi* | \ | \ | MK361031 | \ | \ |
| *Haemaphysalis muhsamae* | NL167-X2 | MT586105 | MN032116 | MT646137 | \ |
| *Haemaphysalis nepalensis* | DQ12 | \ | KY741957 | \ | \ |
| *Haemaphysalis obesa* | \ | \ | KC170732 | \ | \ |
| *Haemaphysalis parva* | \ | \ | MT229176 | MT230039 | \ |
| *Haemaphysalis pentalagi* | Hpen-B | \ | AB819219 | \ | [5] |
| *Haemaphysalis phasiana* | \ | \ | AB819220 | \ | [5] |
| *Haemaphysalis punctaleachi* | \ | \ | KF547994 | \ | [16] |
| *Haemaphysalis punctata* | \ | MF002576 | MF002566 | MH532298 | \ |
| *Haemaphysalis qinghaiensis* | TZ7 | \ | MF629849 | MF981066 | [17] |
| *Haemaphysalis shimoga* | \ | \ | KC170730 | \ | [1] |
| *Haemaphysalis spinigera* | \ | \ | MH044720 | \ | \ |
| *Haemaphysalis spinulosa* | \ | KJ613638 | KJ613637 | \ | [18] |
| *Haemaphysalis sulcata* | \ | \ | MN860530 | MH532303 | \ |
| *Haemaphysalis verticalis* | \ | \ | \ | KY488644 | \ |
| *Haemaphysalis wellingtoni* | \ | \ | MG865746 | \ | [19] |
| *Haemaphysalis yeni* | HY-B | \ | AB819223 | \ | [5] |
| *Haemaphysalis sp.* | \ | \ | \ | MN686567 | \ |
| *Hyalomma aegyptium* | Hyag1 | KT391022 | KT391050 | KT989614 | [20] |
| *Hyalomma albiparmatum* | \ | \ | KU130412 | \ | [21] |
| *Hyalomma anatolicum* | \ | \ | \ | MN728997 | [22] |
| *Hyalomma arabica* | \ | \ | KU130418 | \ | [21] |
| *Hyalomma asiaticum* 1 | XJ191 | KF583617 | KC203350 | KF583578 | \ |
| *Hyalomma asiaticum* 2 | Hyas-2018 | CM023494 | CM023494 | CM023494 | [23] |
| *Hyalomma asiaticum* 3 | IM043 | KF583623 | JX051082 | JX051139 | [24, 25] |
| *Hyalomma detritum* | XJ142 | KF583604 | KC203346 | KF583580 | [24] |
| *Hyalomma dromedarii* | \ | MH094489 | \ | MH094470 | \ |
| *Hyalomma excavatum* | \ | \ | MT229184 | MT230049 | \ |
| *Hyalomma glabrum* | \ | \ | \ | MK551196 | \ |
| *Hyalomma hussaini* | \ | \ | \ | MN728999 | [23] |
| *Hyalomma impeltatum* | \ | MH094500 | \ | \ | [26] |
| *Hyalomma impressum* | \ | \ | KU130437 | \ | [21] |
| *Hyalomma isaaci* | \ | \ | KU130439 | \ | [21] |
| *Hyalomma kumari* | \ | \ | KU130443 | \ | [21] |
| *Hyalomma lusitanicum* | \ | \ | MG855659 | MG855655 | [27] |
| *Hyalomma marginatum* | ERU1 | \ | MT229186 | MT230053 | \ |
| *Hyalomma marginatum sensu lato* | \ | \ | MK058363 | \ | \ |
| *Hyalomma nitidum* | \ | \ | \ | MN244336 | [28] |
| *Hyalomma punt* | \ | \ | KU130454 | \ | [21] |
| *Hyalomma rhipicephaloides* | \ | \ | KU130455 | \ | [21] |
| *Hyalomma rufipes* | \ | \ | \ | MN601294 | [29] |
| *Hyalomma schulzei* 1 | \ | \ | \ | KP241853 | [30] |
| *Hyalomma schulzei* 2 | \ | \ | \ | KP219871 | [30] |
| *Hyalomma schulzei* 3 | \ | \ | \ | KR075985 | \ |
| *Hyalomma schulzei* 4 | \ | \ | KU130632 | KU130467 | [21] |
| *Hyalomma schulzei* 5 | \ | \ | \ | KU130631 | [21] |
| *Hyalomma somalicum* | \ | \ | KU130473 | \ | [21] |
| *Hyalomma truncatum* | Htrun4 | \ | KU130477 | KU130642 | [21] |
| *Hyalomma sp.* | AN84803 | MH061013 | MH061007 | \ | [31] |
| *Rhipicephalus appendiculatus* | \ | \ | \ | MN756039 | [32] |
| *Rhipicephalus bergeoni* | \ | KX377408 | MN944884[ | \ | [33, 34] |
| *Rhipicephalus bursa* | \ | KF219719 | KF219729 | KF219740 | [35] |
| *Rhipicephalus camicasi* | \ | KU746974 | KU746973 | \ | \ |
| *Rhipicephalus cf. camicasi* | \ | MH094507 | \ | MH094482 | \ |
| *Rhipicephalus complanatus* | NL219X-4 | MT586096 | MK894249 | MT646130 | \ |
| *Rhipicephalus compositus* | \ | \ | \ | AF132834 | [36] |
| *Rhipicephalus congolensis* | NL244X-4 | MT586100 | MK894254 | MT646133 | \ |
| *Rhipicephalus duttoni* | A3012 | MF425966 | MF425976 | MF425991 | [37] |
| *Rhipicephalus evertsi* | A3010 | MF425965 | MF425975 | MF425990 | \ |
| *Rhipicephalus glabroscutatus* | \ | \ | \ | MN453672 | \ |
| *Rhipicephalus guilhoni* | \ | KC243814 | KC243854 | KC243900 | [38] |
| *Rhipicephalus haemaphysaloides* | \ | \ | MH208550 | MH208697 | [12] |
| *Rhipicephalus jeanneli* | \ | \ | KY413798 | \ | [39] |
| *Rhipicephalus kochi* | \ | AF150051 | \ | \ | [40] |
| *Rhipicephalus leporis* | \ | \ | \ | KX757917 | [41] |
| *Rhipicephalus longus* | \ | \ | KY413796 | \ | [39] |
| *Rhipicephalus maculatus* | ST61 | \ | KP858499 | KP862678 | [42] |
| *Rhipicephalus muhsamae* | \ | KC243829 | KC243868 | KC243922 | [38] |
| *Rhipicephalus praetextatus* | \ | \ | \ | MF361717 | [43] |
| *Rhipicephalus pravus* | \ | MF361790 | \ | MF361722 | [43] |
| *Rhipicephalus pulchellus* | pulchellusM5 | KY676842 | KY676821 | KY678134 | [44] |
| *Rhipicephalus pumilio* | \ | \ | \ | AY008684 | [36] |
| *Rhipicephalus punctatus* | \ | \ | \ | AF132838 | [36] |
| *Rhipicephalus pusillus* | CR1563 | MF425936 | MF425983 | MF425999 | [37] |
| *Rhipicephalus rossicus* | \ | \ | KX793733 | KX757899 | [41] |
| *Rhipicephalus sanguineus* sensu lato | CR1551 | MF425935 | MF425982 | MF425997 | [37] |
| *Rhipicephalus sanguineus* | Rsan-ML2 | \ | MG855660 | MG855656 | \ |
| *Rhipicephalus senegalensis* | GB3060 | MF425973 | MF425985 | MF426005 | [37] |
| *Rhipicephalus simpsoni* | \ | \ | MK941214 | \ | [45] |
| *Rhipicephalus simus* | RAS3062 | MF425938 | MF425949 | MF425986 | [39] |
| *Rhipicephalus sulcatus* | Rsul4H | KU568504 | \ | KU568514 | [46] |
| *Rhipicephalus turanicus* | \ | MN945345 | MN944872 | MT230062 | [34] |
| *Rhipicephalus zambeziensis* | \ | \ | \ | AY008683 | [36] |
| *Rhipicephalus zumpti* | \ | AF150016 | \ | \ | [40] |

*As a synonym of *Haemaphysalis sulcata* according to Filippova (1997) [47] and Camicas et al. (1998) [48] as well as Chen & Liu (2020) [49].

References

1. Arthan W, Sumrandee C, Hirunkanokpun S, Kitthawee S, Baimai V, Trinachartvanit W, et al. Detection of Coxiella-like endosymbiont in *Haemaphysalis* tick in Thailand. Ticks Tick-Borne Dis. 2015;6:63–8.
2. Evans ML, Egan S, Irwin PJ, Oskam CL. Automatic Barcode Gap Discovery reveals large COI intraspecific divergence in Australian Ixodidae. Zootaxa. 2019;4656:393–6.
3. Thompson AT, Dominguez K, Cleveland CA, Dergousoff SJ, Doi K, Falco RC, et al. Molecular characterization of *Haemaphysalis* species and a molecular genetic key for the identification of *Haemaphysalis* of North America. Front Vet Sci. 2020;7:141.
4. Hornok S, Flaisz B, Takács N, Kontschán J, Csörgő T, Csipak Á, et al. Bird ticks in Hungary reflect western, southern, eastern flyway connections and two genetic lineages of *Ixodes frontalis* and *Haemaphysalis concinna*. Parasit Vectors. 2016;9:101.
5. Takano A, Fujita H, Kadosaka T, Takahashi M, Yamauchi T, Lshiguro F, et al. Construction of a DNA database for ticks collected in Japan: application of molecular identification based on the mitochondrial 16S rDNA gene. Med Entomol Zool. 2014;65:13–21.
6. Norris DE, Klompen JSH, Black WC. Comparison of the mitochondrial 12S and 16S ribosomal DNA genes in resolving phylogenetic relationships among hard ticks (Acari: Ixodidae). Ann Entomol Soc Am.1999;1:117–29.
7. Black WC, Piesman J. Phylogeny of hard- and soft-tick taxa (Acari: Ixodida) based on mitochondrial 16S rDNA sequences. Proc Natl Acad Sci U S A. 1994;91:10034–8.
8. Chen X, Yu Z, Guo L, Li L, Meng H, Wang D, et al. Life cycle of *Haemaphysalis doenitzi* (Acari: Ixodidae) under laboratory conditions and its phylogeny based on mitochondrial 16S rDNA. Exp Appl Acarol. 2012;56:143–50.
9. Chen X, Xu S, Yu Z, Guo L, Yang S, Liu L, et al. Multiple lines of evidence on the genetic relatedness of the parthenogenetic and bisexual *Haemaphysalis longicornis* (Acari: Ixodidae). Infect Genet Evol. 2014;21:308–14.
10. Ernieenor FCL, Ernna G, Mariana A. Phenotypic and genotypic identification of hard ticks of the genus *Haemaphysalis* (Acari: Ixodidae) in Peninsular Malaysia. Exp Appl Acarol. 2017;71:387–400.
11. Binetruy F, Chevillon C, de Thoisy B, Garnier S, Duron O. Survey of ticks in French Guiana. Ticks Tick-Borne Dis. 2019;10:77–85.
12. Li LH, Zhang Y, Wang JZ, Li XS, Yin SQ, Zhu D, et al. High genetic diversity in hard ticks from a China-Myanmar border county. Parasit Vectors. 2018;11:469.
13. Wattanamethanont J, Kaewthamasorn M, Tiawsirisup S. Natural infection of questing ixodid ticks with protozoa and bacteria in Chonburi Province, Thailand. Ticks Tick-Borne Dis. 2018;9:749–58.
14. Blanco MB, Elfawal MA, Durden LA, Beati L, Xu G, Godfrey LR, et al. Genetic diversity of ixodid ticks parasitizing eastern mouse and dwarf lemurs in Madagascar, with descriptions of the larva, nymph, and male of *Ixodes lemuris* (Acari: Ixodidae). J Parasitol. 2013;99:11–8.
15. Seo MG, Kwon OD, Kwak D. Genotypic analysis of piroplasms and associated pathogens from ticks infesting cattle in Korea. Microorganisms. 2020;8:728.
16. Wang Y-Z, Mu L-M, Zhang K, Yang M-H, Zhang L, Du J-Y, et al. A broad-range survey of ticks from livestock in Northern Xinjiang: changes in tick distribution and the isolation of *Borrelia burgdorferi sensu stricto*. Parasit Vectors. 2015;8:449.
17. Liu X, Chen Z, Ren Q, Luo J, Xu X, Wu F, et al. Genetic diversity of *Haemaphysalis qinghaiensis* (Acari: Ixodidae) in western China. Exp Appl Acarol. 2018;74:427–41.
18. Halajian A, Palomar AM, Portillo A, Heyne H, Luus-Powell WJ, Oteo JA. Investigation of *Rickettsia*, *Coxiella burnetii* and *Bartonella* in ticks from animals in South Africa. Ticks Tick-Borne Dis. 2016;7:361–6.
19. Trinachartvanit W, Maneewong S, Kaenkan W, Usananan P, Baimai V, Ahantarig A. Coxiella-like bacteria in fowl ticks from Thailand. Parasit Vectors. 2018;11:670.
20. Roth A, Akad F, Zonstein I, King R, Orshan L, Erster O. Molecular characterization of six *Hyalomma* species using mitochondrial markers. Ticks Tick-Borne Dis. 2019;10:911–7.
21. Sands AF, Apanaskevich DA, Matthee S, Horak IG, Harrison A, Karim S, et al. Effects of tectonics and large scale climatic changes on the evolutionary history of *Hyalomma* ticks. Mol Phylogenet Evol. 2017;114:153–65.
22. Ghafar A, Gasser RB, Rashid I, Ghafoor A, Jabbar A. Exploring the prevalence and diversity of bovine ticks in five agro-ecological zones of Pakistan using phenetic and genetic tools. Ticks Tick-Borne Dis. 2020;11:101472.
23. Jia N, Wang J, Shi W, Du L, Sun Y, Zhan W, et al. Large-scale comparative analyses of tick genomes elucidate their genetic diversity and vector capacities. Cell. 2020;182:1328–40.
24. Lv J, Wu S, Zhang Y, Chen Y, Feng C, Yuan X, et al. Assessment of four DNA fragments (COI, 16S rDNA, ITS2, 12S rDNA) for species identification of the Ixodida (Acari: Ixodida). Parasit Vectors. 2014;7:93.
25. Lv J, Wu S, Zhang Y, Zhang T, Feng C, Jia G, et al. Development of a DNA barcoding system for the Ixodida (Acari: Ixodida). Mitochondrial DNA. 2014;25:142–9.
26. Chandra S, Smith K, Alanazi AD, Alyousif MS, Emery D, Slapeta J. *Rhipicephalus sanguineus sensu lato* from dogs and dromedary camels in Riyadh, Saudi Arabia: low prevalence of vector-borne pathogens in dogs detected using multiplexed tandem PCR panel. Folia Parasitol. 2019;66:007.
27. Hornok S, Grima A, Takács N, Szekeres S, Kontschán J. First records and molecular-phylogenetic analyses of three tick species (*Ixodes kaiseri, Hyalomma lusitanicum* and *Ornithodoros coniceps*) from Malta. Ticks Tick-Borne Dis. 2020;11:101379.
28. Schulz A, Karger A, Bettin B, Eisenbarth A, Sas MA, Silaghi C, et al. Molecular discrimination of *Hyalomma* tick species serving as reservoirs and vectors for Crimean-Congo hemorrhagic fever virus in sub-Saharan Africa. Ticks Tick-Borne Dis. 2020;11:101382.
29. Onyiche TE, Răileanu C, Tauchmann O, Fischer S, Vasić A, Schäfer M, et al. Prevalence and molecular characterization of ticks and tick-borne pathogens of one-humped camels (*Camelus dromedarius*) in Nigeria. Parasit Vectors. 2020;13:428.
30. Hosseini-Chegeni A, Hosseini R, Telmadarraiy Z, Abdigoudarzi M. The Iranian *Hyalomma* (Acari: Ixodidae) with molecular evidences to understand taxonomic status of species complexes. Persian J Acarol. 2019;8:291–308.
31. Hoffman T, Lindeborg M, Barboutis C, Erciyas-Yavuz K, Evander M, Fransson T, et al. Alkhurma hemorrhagic fever virus RNA in *Hyalomma rufipes* ticks infesting migratory birds, Europe and Asia Minor. Emerg Infect Dis. 2018;24:879–82.
32. Obara I, Githaka N, Nijhof A, Krücken J, Nanteza A, Odongo D, et al. The *Rhipicephalus appendiculatus* tick vector of *Theileria parva* is absent from Cape buffalo (*Syncerus caffer*) populations and associated ecosystems in northern Uganda. Parasitol Res. 2020;119:2363–7.
33. Kumsa B, Laroche M, Almeras L, Mediannikov O, Raoult D, Parola P. Morphological, molecular and MALDI-TOF mass spectrometry identification of ixodid tick species collected in Oromia, Ethiopia. Parasitol Res. 2016;115:4199–210.
34. Bakkes DK, Chitimia-Dobler L, Matloa D, Oosthuysen M, Mumcuoglu KY, Mans BJ, et al. Integrative taxonomy and species delimitation of *Rhipicephalus turanicus* (Acari: Ixodida: Ixodidae). Int J Parasitol. 2020;50:577–94.
35. Erster O, Roth A, Wolkomirsky R, Leibovich B, Shkap V. Comparative analysis of mitochondrial markers from four species of *Rhipicephalus* (Acari: Ixodidae). Vet Parasitol. 2013;198:364–70.
36. Murrell A, Campbell NJ, Barker SC. Phylogenetic analyses of the rhipicephaline ticks indicate that the genus *Rhipicephalus* is paraphyletic. Mol Phylogenet Evol. 2000;16:1–7.
37. Coimbra-Dores MJ, Maia-Silva M, Marques W, Oliveira AC, Rosa F, Dias D. Phylogenetic insights on Mediterranean and Afrotropical *Rhipicephalus* species (Acari: Ixodida) based on mitochondrial DNA. Exp Appl Acarol. 2018;75:107–28.
38. Dantas-Torres F, Latrofa MS, Annoscia G, Giannelli A, Parisi A, Otranto D. Morphological and genetic diversity of *Rhipicephalus sanguineus* sensu lato from the New and Old Worlds. Parasit Vectors. 2013;6:213.
39. Chitimia-Dobler L, Langguth J, Pfeffer M, Kattner S, Küpper T, Friese D, et al. Genetic analysis of *Rhipicephalus sanguineus* sensu lato ticks parasites of dogs in Africa north of the Sahara based on mitochondrial DNA sequences. Vet Parasitol. 2017;239:1–6.
40. Beati L, Keirans JE. Analysis of the systematic relationships among ticks of the genera *Rhipicephalus* and *Boophilus* (Acari: Ixodidae) based on mitochondrial 12S ribosomal DNA gene sequences and morphological characters. J Parasitol. 2001;87:32-48.
41. Hornok S, Sándor AD, Tomanović S, Beck R, D'Amico G, Kontschán J, et al. East and west separation of *Rhipicephalus sanguineus* mitochondrial lineages in the Mediterranean Basin. Parasit Vectors. 2017;10:39.
42. Mwamuye MM, Kariuki E, Omondi D, Kabii J, Odongo D, Masiga D, et al. Novel rickettsia and emergent tick-borne pathogens: A molecular survey of ticks and tick-borne pathogens in Shimba Hills National Reserve, Kenya. Ticks Tick-Borne Dis. 2017;8:208–18.
43. Kanduma EG, Bishop RP, Githaka NW, Skilton RA, Heyne H, Mwacharo JM. Mitochondrial and nuclear multilocus phylogeny of *Rhipicephalus* ticks from Kenya. Mol Phylogenet Evol. 2019;140:106579.
44. Duron O, Binetruy F, Noël V, Cremaschi J, McCoy KD, Arnathau C, et al. Evolutionary changes in symbiont community structure in ticks. Mol Ecol. 2017;26:2905–21.
45. Shuaib YA , Elhag AMW, Brima YA, Abdalla MA, Bakiet AO, Mohmed-Noor SE, et al. Ixodid tick species and two tick-borne pathogens in three areas in the Sudan. Parasitol Res. 2020;119:385–94.
46. Zúquete ST, Coelho J, Rosa F, Vaz Y, Cassamá B, Padre L, et al. Tick (Acari: Ixodidae) infestations in cattle along Geba River basin in Guinea-Bissau. Ticks Tick-Borne Dis. 2017;8:161–9.
47. Filippova NA. Ixodid ticks of subfamily Amblyomminae. Fauna of Russia and neighboring countries. St. Petersburg: Nauka Publishing House.1997;436 pp (In Russian).
48. Camicas JL, Hervy JP, Adam F, Morel PC. Les Tiques du Monde (Acarida, Ixodida). Nomenclature, Stades Décrits, Hôtes, Répartition. Orstom, Paris, France. 1998;300 pp(In French).
49. Chen C, Liu JZ. Recent progress in tick taxonomy and a global list of tick species. Chin J Appl Entomol. 2020;57:1009–45 (In Chinse with English abstract).
