# Supplementary material for: Records of three mammal tick species parasitizing an atypical host, the multi-ocellated racerunner lizard, in arid regions of Xinjiang, China
Source: Parasit Vectors. 2021 Mar 4;14:135. doi: 10.1186/s13071-021-04639-z (PMC7931338; doi:10.1186/s13071-021-04639-z)
Supplement: Supplementary file 4 — Additional file 4: Table S4. List of the host, origin, tick species, haplotype number, and GenBank accession numbers of the ticks obtained from this study and used for median-joining network presented in Figs. 2, 3, 4, 5. [file 13071_2021_4639_MOESM4_ESM.docx]

Table S4. List of the host, origin, tick species, haplotype number and GenBank accession numbers of the ticks obtained from this study and used for median-joining network presented in Figs. 2−5.

| Host | Origin code | Tick species | Gene | Alignment length (bp) | Haplotype number | GenBank accession number |
| --- | --- | --- | --- | --- | --- | --- |
| *E. multiocellata* | P3 | *H. sulcata* | *16S rRNA* | 266 | Q1 | MN267476 |
| *E. multiocellata* | P1 | *H. sulcata* | *16S rRNA* | 266 | Q2 | MN267477 |
| *E. multiocellata* | P1 | *H. sulcata* | *COI* | 550 | Q3 | MT237703 |
| *E. multiocellata* | P3 | *H. sulcata* | *COI* | 550 | Q4 | MT237702 |
| *E. multiocellata* | P1 | *H. sulcata* | *COI* | 550 | Q5 | MT237701 |
| *E. multiocellata* | P2 | *H. sulcata* | *COI* | 550 | Q6 | MT237700 |
| *E. multiocellata* | P3 | *H. sulcata* | *COI* | 550 | Q7 | MT246592 |
| *E. multiocellata* | P3 | *H. sulcata* | *COI* | 550 | Q8 | MT237698 |
| *E. multiocellata* | P1 | *H. sulcata* | *COI* | 550 | Q9 | MT237699 |
| *E. multiocellata* | P1 | *R. turanicus* | *COI* | 496 | Z1 | MT237709 |
| *E. multiocellata* | P3 | *R. turanicus* | *COI* | 496 | Z1 | MT237704 |
| *E. multiocellata* | P1 | *R. turanicus* | *COI* | 496 | Z1 | MT237705 |
| *E. multiocellata* | P1 | *Hy. asiaticum* | *COI* | 418 | C1 | MT237681 |
| *E. multiocellata* | P1 | *Hy. asiaticum* | *COI* | 418 | C1 | MT237654 |
| *E. multiocellata* | P3 | *Hy. asiaticum* | *COI* | 418 | C1 | MT237661 |
| *E. multiocellata* | P1 | *Hy. asiaticum* | *COI* | 418 | C1 | MT237667 |
| *E. multiocellata* | P1 | *Hy. asiaticum* | *COI* | 418 | C1 | MT237672 |
| *E. multiocellata* | P1 | *Hy. asiaticum* | *COI* | 418 | C1 | MT237675 |
| *E. multiocellata* | P3 | *Hy. asiaticum* | *COI* | 418 | C1 | MT237676 |
| *E. multiocellata* | P3 | *Hy. asiaticum* | *COI* | 418 | C1 | MT237653 |
| Hedgehog | P5 | *R. turanicus* | *COI* | 496 | Z1 | MT237706 |
| Hedgehog | P5 | *R. turanicus* | *COI* | 496 | Z1 | MT237707 |
| Hedgehog | P5 | *R. turanicus* | *COI* | 496 | Z1 | MT237708 |
| Hedgehog | P5 | *Hy. asiaticum* | *COI* | 418 | C1 | MT237655 |
| Hedgehog | P5 | *Hy. asiaticum* | *COI* | 418 | C1 | MT237657 |
| Hedgehog | P5 | *Hy. asiaticum* | *COI* | 418 | C3 | MT237658 |
| Hedgehog | P5 | *Hy. asiaticum* | *COI* | 418 | C3 | MT237659 |
| Hedgehog | P5 | *Hy. asiaticum* | *COI* | 418 | C1 | MT237660 |
| Hedgehog | P5 | *Hy. asiaticum* | *COI* | 418 | C1 | MT237663 |
| Hedgehog | P5 | *Hy. asiaticum* | *COI* | 418 | C1 | MT237664 |
| Hedgehog | P5 | *Hy. asiaticum* | *COI* | 418 | C1 | MT237665 |
| Hedgehog | P5 | *Hy. asiaticum* | *COI* | 418 | C1 | MT237668 |
| Hedgehog | P5 | *Hy. asiaticum* | *COI* | 418 | C1 | MT237669 |
| Hedgehog | P5 | *Hy. asiaticum* | *COI* | 418 | C1 | MT237670 |
| Hedgehog | P5 | *Hy. asiaticum* | *COI* | 418 | C1 | MT237671 |
| Hedgehog | P5 | *Hy. asiaticum* | *COI* | 418 | C1 | MT237673 |
| Hedgehog | P5 | *Hy. asiaticum* | *COI* | 418 | C1 | MT237674 |
| Hedgehog | P5 | *Hy. asiaticum* | *COI* | 418 | C1 | MT237677 |
| Hedgehog | P5 | *Hy. asiaticum* | *COI* | 418 | C1 | MT237679 |
| Hedgehog | P5 | *Hy. asiaticum* | *COI* | 418 | C1 | MT237680 |
| Hedgehog | P5 | *Hy. asiaticum* | *COI* | 418 | C1 | MT237682 |
| Hedgehog | P5 | *Hy. asiaticum* | *COI* | 418 | C1 | MT237683 |
| Hedgehog | P5 | *Hy. asiaticum* | *COI* | 418 | C1 | MT237684 |
| Hedgehog | P5 | *Hy. asiaticum* | *COI* | 418 | C1 | MT237685 |
| Hedgehog | P5 | *Hy. asiaticum* | *COI* | 418 | C1 | MT237686 |
| Hedgehog | P5 | *Hy. asiaticum* | *COI* | 418 | C4 | MT237688 |
| Hedgehog | P5 | *Hy. asiaticum* | *COI* | 418 | C4 | MT237690 |
| Hedgehog | P5 | *Hy. asiaticum* | *COI* | 418 | C3 | MT237691 |
| Hedgehog | P5 | *Hy. asiaticum* | *COI* | 418 | C1 | MT237692 |
| Hedgehog | P5 | *Hy. asiaticum* | *COI* | 418 | C1 | MT237694 |
| Hedgehog | P5 | *Hy. asiaticum* | *COI* | 418 | C1 | MT237695 |
| Hedgehog | P5 | *Hy. asiaticum* | *COI* | 418 | C2 | MT237666 |
| Hedgehog | P5 | *Hy. asiaticum* | *COI* | 418 | C5 | MT237689 |
| Hedgehog | P5 | *Hy. asiaticum* | *COI* | 418 | C2 | MT237693 |
| Hedgehog | P5 | *Hy. asiaticum* | *COI* | 418 | C1 | MT240263 |
| Hedgehog | P5 | *Hy. asiaticum* | *COI* | 418 | C1 | MT240264 |

P1, P2, P3, and P5 correspond to those in Table 1
