# Supplementary material for: Records of three mammal tick species parasitizing an atypical host, the multi-ocellated racerunner lizard, in arid regions of Xinjiang, China
Source: Parasit Vectors. 2021 Mar 4;14:135. doi: 10.1186/s13071-021-04639-z (PMC7931338; doi:10.1186/s13071-021-04639-z)
Supplement: Supplementary file 5 — Additional file 5: Table S5. Accession numbers for eight 16S rRNA gene sequences of Haemaphysalis sulcata downloaded from GenBank and used for the median-joining network presented in Fig. 2. [file 13071_2021_4639_MOESM5_ESM.docx]

Table S5. Accession numbers for 8 *16S rRNA* gene sequences of *Haemaphysalis sulcata* downloaded from GenBank and used for the median-joining network presented in Fig. 2.

| GenBank accession number | Species | Origin/Host | References |
| --- | --- | --- | --- |
| KX576650 | *H. sulcata* | France/Mouflon | [1] |
| MN860530 | *H. sulcata* | China: Xinjiang/free | \ |
| MT799946 | *H. sulcata* | Pakistan/goat | [2] |
| MT799949 | *H. sulcata* | Pakistan/sheep | [2] |
| MT799947 | *H. sulcata* | Pakistan/goat | [2] |
| MT799948 | *H. sulcata* | Pakistan/goat | [2] |
| L34308 | *H. sulcata* | \ | [3] |
| MN860531 | *H. sulcata* | China: Xinjiang/free | \ |

References

1. Grech-Angelini S, Stachurski F, Lancelot R, Boissier J, Allienne JF, Marco S, et al. Ticks (Acari: Ixodidae) infesting cattle and some other domestic and wild hosts on the French Mediterranean island of Corsica. Parasit Vectors. 2016;9:582.
2. Ghafar A, Khan A, Cabezas-Cruz A, Gauci CG, Niaz S, Ayaz S, et al. An assessment of the molecular diversity of ticks and tick-borne microorganisms of small ruminants in Pakistan. Microorganisms. 2020;8:1428.
3. Black WC, Piesman J. Phylogeny of hard- and soft-tick taxa (Acari: Ixodida) based on mitochondrial 16S rDNA sequences. Proc Natl Acad Sci U S A. 1994;91:10034–8.
