# Supplementary material for: Records of three mammal tick species parasitizing an atypical host, the multi-ocellated racerunner lizard, in arid regions of Xinjiang, China
Source: Parasit Vectors. 2021 Mar 4;14:135. doi: 10.1186/s13071-021-04639-z (PMC7931338; doi:10.1186/s13071-021-04639-z)
Supplement: Supplementary file 6 — Additional file 6: Table S6. Accession numbers for 9 COI gene sequences of Haemaphysalis sulcata downloaded from GenBank and used for the median-joining network presented in Fig. 3. [file 13071_2021_4639_MOESM6_ESM.docx]

| GenBank accession number | Origin/Host | References |
| --- | --- | --- |
| MT800321 | Pakistan/sheep | [1] |
| MT800320 | Pakistan/goat | [1] |
| MT800319 | Pakistan/goat | [1] |
| MH532303 | Iran/livestock | [2] |
| MH532302 | Iran/livestock | [2] |
| MH532301 | Iran/livestock | [2] |
| MH532299 | Iran/livestock | [2] |
| JX394188 | Romania | \ |
| JX394189 | Romania | \ |

Table S6. Accession numbers for 9 *COI* gene sequences of *Haemaphysalis sulcata* downloaded from GenBank and used for the median-joining network presented in Fig. 3.

References

1. Ghafar A, Khan A, Cabezas-Cruz A, Gauci CG, Niaz S, Ayaz S, et al. An assessment of the molecular diversity of ticks and tick-Borne microorganisms of small ruminants in Pakistan. Microorganisms. 2020;8:1428.
2. Faghihi F, Hosseini-Chegeni A, Edalat H, Banafshi O, Sedaghat MM. Molecular identification of some *Haemaphysalis* species (Acari: Ixodidae) using mitochondrial and nuclear evidences in parts of Iran. Syst Appl Acarol. 2020;25:809–20.
