# Supplementary material for: Records of three mammal tick species parasitizing an atypical host, the multi-ocellated racerunner lizard, in arid regions of Xinjiang, China
Source: Parasit Vectors. 2021 Mar 4;14:135. doi: 10.1186/s13071-021-04639-z (PMC7931338; doi:10.1186/s13071-021-04639-z)
Supplement: Supplementary file 7 — Additional file 7: Table S7. Accession numbers for 81 COI gene sequences of Rhipicephalus turanicus downloaded from GenBank and used for the median-joining network presented in Fig. 4. [file 13071_2021_4639_MOESM7_ESM.docx]

Table S7. Accession numbers for 81 *COI* gene sequences of *Rhipicephalus turanicus* downloaded from GenBank and used for the median-joining network presented in Fig. 4.

| GenBank accession number | Origin/Host | References |
| --- | --- | --- |
| MW065551 | China: Xinjiang/dog | \ |
| MT800312 | Pakistan/*Ovis aries* | [1] |
| MT800313 | Pakistan /goat | [1] |
| MT800314 | Pakistan/*Ovis aries* | [1] |
| MN689425 | Kazakhstan/ dog | \ |
| MN689420 | Kazakhstan/ cattle | \ |
| MN689410 | Kazakhstan/ cattle | \ |
| MN517834 | China: Xinjiang/horse | [2] |
| MT079206 | Kazakhstan/ cattle | \ |
| MN853166 | Kazakhstan/ cattle | \ |
| MN841462 | Kazakhstan/ dog | \ |
| MN907846 | Kazakhstan/ cattle | \ |
| KY488675 | China: Shandong | [3] |
| KY488674 | China: Shandong | [3] |
| KY488673 | China: Shandong | [3] |
| KY488665 | China: Shandong | [3] |
| KY488663 | China: Shandong | [3] |
| KY488662 | China: Shandong | [3] |
| KY488661 | China: Shandong | [3] |
| KY488641 | China: Shandong | [3] |
| KY488640 | China: Shandong | [3] |
| MH094483 | Saudi Arabia/cattle | [4] |
| KT313122 | Iran/sheep | [5] |
| KT313121 | Iran/sheep | [5] |
| KT313117 | Iran/sheep | [5] |
| MF002581 | China: Xinjiang/sheep | \ |
| MF002580 | China: Xinjiang/sheep | \ |
| MF002579 | China: Xinjiang/sheep | \ |
| MF002578 | China: Xinjiang/sheep | \ |
| MF002577 | China: Xinjiang/sheep | \ |
| KY069271 | China: Xinjiang/dog | \ |
| KX757894 | Croatia/ | [6] |
| KX757891 | Croatia/ | [6] |
| KX757886 | Croatia/ | [6] |
| KX757884 | Croatia/ | [6] |
| KY606303 | Albania/sheep | [7] |
| KY606302 | Albania/sheep | [7] |
| KY606301 | Albania/sheep | [7] |
| KY606300 | Albania/sheep | [7] |
| KY606299 | Albania/sheep | [7] |
| KY606298 | Albania/sheep | [7] |
| KY606297 | Albania/sheep | [7] |
| KY606296 | China/sheep | [7] |
| KY606295 | China/sheep | [7] |
| KY606294 | China/sheep | [7] |
| KY606291 | China/sheep | [7] |
| KY606293 | China/sheep | [7] |
| KY606292 | China/sheep | [7] |
| KY606290 | China/sheep | [7] |
| KY606289 | China/sheep | [7] |
| KY606288 | China/sheep | [7] |
| KY606287 | China/sheep | [7] |
| KU880593 | China | \ |
| KU880592 | China | \ |
| KU880591 | China | \ |
| KU880590 | China | \ |
| KU880576 | China | \ |
| KU880575 | China | \ |
| KU880574 | China | \ |
| KU880564 | China | \ |
| KU880563 | China | \ |
| JQ737086 | China: Xinjiang/sheep | [8] |
| KU364306 | China: Xinjiang/free | [9] |
| KU364303 | China: Xinjiang/free | [9] |
| KU364304 | China: Xinjiang/free | [9] |
| KU364305 | China: Xinjiang/free | [9] |
| KM235719 | Iraq | \ |
| KM235718 | Iraq | \ |
| KM235717 | Iraq | \ |
| KF688138 | China: Xinjiang/sheep | [10] |
| KF688136 | China: Xinjiang/sheep | [10] |
| KF688137 | China: Xinjiang/sheep | [10] |
| KF219750 | Israel/cattle | [11] |
| KF219747 | Israel/cattle | [11] |
| KF219748 | Israel/cattle | [11] |
| KF219749 | Israel/cattle | [11] |
| KF251021 | Israel/cattle | [11] |
| KF251020 | Israel/cattle | [11] |
| KF251019 | Israel/cattle | [11] |
| MW018427 | Bulgaria/*Bubo bubo* | \ |
| MK038884 | Bulgaria/*Bubo bubo* | \ |

References

1. Ghafar A, Khan A, Cabezas-Cruz A, Gauci CG, Niaz S, Ayaz S, et al. An assessment of the molecular diversity of ticks and tick-borne microorganisms of small ruminants in Pakistan. Microorganisms. 2020;8:1428.
2. Song R, Ma Y, Hu Z, Li Y, Li M, Wu L, et al. MaxEnt modeling of *Dermacentor marginatus* (Acari: Ixodidae) distribution in Xinjiang, China. J Med Entomol. 2020;57:1659–67.
3. Zhang R, Zhao A, Wang X, Zhang Z. Diversity of tick species on domestic animals in Shandong Province, China, using DNA barcoding. Exp Appl Acarol. 2017;73:79–89.
4. Chandra S, Smith K, Alanazi AD, Alyousif MS, Emery D, Slapeta J. *Rhipicephalus sanguineus sensu lato* from dogs and dromedary camels in Riyadh, Saudi Arabia: low prevalence of vector-borne pathogens in dogs detected using multiplexed tandem PCR panel. Folia Parasitol. 2019;66:007.
5. Hosseini-Chegeni A, Nasrabadi M, Sadat S HA, Oshaghi MA, Lotfi A, Telmadarraiy Z, et al. Molecular identification of *Rhipicephalus* species (Acari: Ixodidae) parasitizing livestock from Iran. Mitochondrial DNA A. 2019;30:448–56.
6. Hornok S, Sándor AD, Tomanović S, Beck R, D'Amico G, Kontschán J, et al. East and west separation of *Rhipicephalus sanguineus* mitochondrial lineages in the Mediterranean Basin. Parasit Vectors. 2017;10:39.
7. Li HY, Zhao SS, Hornok S, Farkas R, Guo LP, Chen CF, et al. Morphological and molecular divergence of *Rhipicephalus turanicus* tick from Albania and China. Exp Appl Acarol. 2017;73:493–9.
8. Gou H, Xue H, Yin H, Luo J, Sun X. Molecular characterization of hard ticks by cytochrome c oxidase subunit 1 sequences. Korean J Parasitol. 2018;56:583–8.
9. Wang AD. Identification of vector species and investigation of diseases in Alashankou Port. Master Dissertation. Shihezi, Xinjiang, China: Shihezi University. 2016 (In Chinese with English abstract).
10. Wei QQ, Guo LP, Wang AD, Mu LM, Zhang K, Chen CF, et al. The first detection of *Rickettsia aeschlimannii* and *Rickettsia massiliae* in *Rhipicephalus turanicus* ticks, in northwest China. Parasit Vectors. 2015;8:631.
11. Erster O, Roth A, Wolkomirsky R, Leibovich B, Shkap V. Comparative analysis of mitochondrial markers from four species of *Rhipicephalus* (Acari: Ixodidae). Vet Parasitol. 2013;198:364–70.
