# Supplementary material for: Records of three mammal tick species parasitizing an atypical host, the multi-ocellated racerunner lizard, in arid regions of Xinjiang, China
Source: Parasit Vectors. 2021 Mar 4;14:135. doi: 10.1186/s13071-021-04639-z (PMC7931338; doi:10.1186/s13071-021-04639-z)
Supplement: Supplementary file 8 — Additional file 8: Table S8. Accession numbers for 104 COI gene sequences of Hyalomma asiaticum downloaded from GenBank and used for the median-joining network presented in Fig. 5. [file 13071_2021_4639_MOESM8_ESM.docx]

Table S8. Accession numbers for 104 *COI* gene sequences of *Hyalomma asiaticum* downloaded from GenBank and used for the median-joining network presented in Fig. 5.

| GenBank accession number | Origin/Host | Haplotype number | References |
| --- | --- | --- | --- |
| MN961479 | Kazakhstan/camel | C1 | \ |
| MN907845 | Kazakhstan/cattle | C1 | \ |
| MN892553 | Kazakhstan/free | C1 | \ |
| MK757583 | China: Xinjiang/camel | C1 | [1] |
| MK292004 | China: Gansu/camel | C1 | [2] |
| MK292001 | China: Gansu/camel | C1 | [2] |
| MK292000 | China: Gansu/camel | C1 | [2] |
| MF101818 | China: Xinjiang/Sheep | C1 | [3] |
| KU880598 | China: Xinjiang | C1 | \ |
| KU880580 | China: Xinjiang | C1 | \ |
| KU364335 | China: Xinjiang | C1 | [4] |
| KU364334 | Kazakhstan | C1 | [5] |
| KU364332 | Kazakhstan | C1 | [5] |
| KU364324 | Kazakhstan | C1 | [5] |
| KU364317 | Kazakhstan | C1 | [5] |
| KP219869 | Iran/ No data available | C1 | [6] |
| KP219864 | Iran/ No data available | C1 | [6] |
| KP219860 | Iran/sheep | C1 | [6] |
| KP208951 | Iran/ No data available | C1 | [6] |
| KF527441 | China: Xinjiang/sheep | C1 | [7] |
| KF527440 | China: Xinjiang/sheep | C1 | [7] |
| JX051148 | China: Inner Mongolia/sheep | C1 | [8] |
| JX051147 | China: Inner Mongolia/sheep | C1 | [8] |
| JX051146 | China: Inner Mongolia/sheep | C1 | [8] |
| JX051131 | China: Inner Mongolia/sheep | C1 | [8] |
| JX051132 | China: Inner Mongolia/sheep | C1 | [8] |
| JX051133 | China: Inner Mongolia/sheep | C1 | [8] |
| JX051135 | China: Inner Mongolia/sheep | C1 | [8] |
| JQ737072 | Chia: Gansu/camel | C1 | [9] |
| JQ737071 | Chia: Inner Mongolia | C1 | \ |
| JX051134 | China: Inner Mongolia/sheep | C2 | [8] |
| MN865123 | Kazakhstan/cattle | C6 | \ |
| MN689404 | Kazakhstan/cattle | C6 | \ |
| MK610453 | China: Xinjiang/sheep | C6 | [10] |
| MK307807 | China: Xinjiang/sheep | C6 | [10] |
| MK213074 | China/free | C6 | \ |
| MK213071 | China/camel | C6 | \ |
| MH459386 | China/cattle | C6 | \ |
| MH459384 | China/cattle | C6 | \ |
| KU880606 | China: Xinjiang | C6 | \ |
| KU880605 | China: Xinjiang | C6 | \ |
| KU880603 | China: Xinjiang | C6 | \ |
| KU880587 | China: Xinjiang | C6 | \ |
| KU880570 | China: Xinjiang | C6 | \ |
| KU880569 | China: Xinjiang | C6 | \ |
| KU364337 | China: Xinjiang | C6 | [4] |
| CM023494 | China: Tibet/free | C6 | [11] |
| MH459385 | China/cattle | C7 | \ |
| MF101817 | China: Xinjiang/sheep | C8 | [3] |
| KU880568 | China: Xinjiang | C8 | \ |
| KU364336 | China: Xinjiang | C8 | [4] |
| KX882103 | China: Xinjiang | C9 | \ |
| KX882102 | China: Xinjiang | C10 | \ |
| KX882101 | China: Xinjiang | C11 | \ |
| KX882100 | China: Xinjiang | C12 | \ |
| KU880604 | China: Xinjiang | C13 | \ |
| KU880602 | China: Xinjiang | C14 | \ |
| KU880567 | China: Xinjiang | C14 | \ |
| KU364330 | Kazakhstan | C14 | [5] |
| KU364322 | Kazakhstan | C14 | [5] |
| KU880601 | China: Xinjiang | C15 | \ |
| KU880582 | China: Xinjiang | C15 | \ |
| KU880566 | China: Xinjiang | C15 | \ |
| KU364320 | Kazakhstan | C15 | [5] |
| KU880600 | China: Xinjiang | C16 | \ |
| KU880599 | China: Xinjiang | C17 | \ |
| KU880581 | China: Xinjiang | C17 | \ |
| KU880565 | China: Xinjiang | C17 | \ |
| KU364326 | Kazakhstan | C17 | [5] |
| KU364318 | Kazakhstan | C17 | [5] |
| KU880586 | China: Xinjiang | C18 | \ |
| KU880585 | China: Xinjiang | C19 | \ |
| KU880584 | China: Xinjiang | C20 | \ |
| KU880583 | China: Xinjiang | C21 | \ |
| KU880579 | China: Xinjiang | C22 | \ |
| KU364333 | Kazakhstan | C23 | [5] |
| KU364331 | Kazakhstan | C24 | [5] |
| KU364327 | Kazakhstan | C24 | [5] |
| KU364329 | Kazakhstan | C25 | [5] |
| KU364328 | Kazakhstan | C26 | [5] |
| KU364325 | Kazakhstan | C27 | [5] |
| KU364315 | China: Xinjiang | C27 | [4] |
| KU364323 | Kazakhstan | C28 | [5] |
| KU364321 | Kazakhstan | C29 | [5] |
| KU364319 | Kazakhstan | C30 | [5] |
| KU364314 | China: Xinjiang | C31 | [4] |
| KU364313 | China: Xinjiang | C32 | [4] |
| KP219866 | Iran/Sheep | C35 | [6] |
| KF583578 | China: Xinjiang, Tulufan/sheep | C36 | [8] |
| KC203440 | China: Xinjiang, Tulufan/sheep | C37 | [8] |
| KC203439 | China: Xinjiang, Tulufan/sheep | C38 | [8] |
| JX051150 | China: Inner Mongolia/sheep | C39 | [8] |
| JX051149 | China: Inner Mongolia/sheep | C39 | [8] |
| JX051145 | China: Inner Mongolia/sheep | C40 | [8] |
| JX051144 | China: Inner Mongolia/sheep | C41 | [8] |
| JX051143 | China: Inner Mongolia/sheep | C41 | [8] |
| JX051142 | China: Inner Mongolia/sheep | C42 | [8] |
| JX051141 | China: Inner Mongolia/sheep | C43 | [8] |
| JX051139 | China: Inner Mongolia/sheep | C44 | [8] |
| JX051138 | China: Inner Mongolia/sheep | C45 | [8] |
| JX051137 | China: Inner Mongolia/sheep | C46 | [8] |
| JQ737073 | China: Gansu/camel | C46 | [9] |
| JX051136 | China: Inner Mongolia/sheep | C47 | [8] |
| JQ737070 | China: Xinjiang | C48 | \ |

References

1. Zhou H, Ma Z, Hu T, Bi Y, Mamuti A, Yu R, et al. Tamdy virus in Ixodid ticks infesting Bactrian camels, Xinjiang, China, 2018. Emerg Infect Dis. 2019;25:2136–8.
2. Guo WP, Wang YH, Lu Q, Xu G, Luo Y, Ni X, et al. Molecular detection of spotted fever group rickettsiae in hard ticks, northern China. Transbound Emerg Dis. 2019;66:1587–96.
3. Liu ZQ, Liu YF, Kuermanali N, Wang DF, Chen SJ, Guo HL, et al. Sequencing of complete mitochondrial genomes confirms synonymization of *Hyalomma asiaticum asiaticum* and *kozlovi*, and advances phylogenetic hypotheses for the Ixodidae. PLoS One. 2018;13:e0197524.
4. Wang AD. Identification of vetor species and investigation of diseases in Alashankou Port. 2016. Master Dissertation. Shihezi, Xinjiang, China: Shihezi University. 2016 (In Chinese with English abstract).
5. Xu J, Wang AD, Dai L, Yang J, Luo D, Xu JJ, et al. Species identification of an imported tick sample of *Hyalomma asiaticum asiaticum* by genetic analysis at Alashankou Port, China-Kazakhstan border. Chin J Vet Med. 2016;11:17–9 (In Chinese with English abstract).
6. Hosseini-Chegeni A, Hosseini R, Telmadarraiy Z, Abdigoudarzi M. The Iranian *Hyalomma* (Acari: Ixodidae) with molecular evidences to understand taxonomic status of species complexes. Persian J Acarol. 2019;8:291–308.
7. Chen Z, Li YQ, Ren QY, Luo J, Hu Y, Li K, et al. Morphological characteristics of normal and gynandromorphic *Hyalomma asiaticum* Schulze and Schlottke, 1930. Korean J Parasitol. 2015;53:361–4.
8. Lv J, Wu S, Zhang Y, Zhang T, Feng C, Jia G, et al. Development of a DNA barcoding system for the Ixodida (Acari: Ixodida). Mitochondrial DNA. 2014;25:142–9.
9. Gou H, Xue H, Yin H, Luo J, Sun X. Molecular characterization of hard ticks by cytochrome c oxidase subunit 1 sequences. Korean J Parasitol. 2018;56:583–8.
10. Jiang MM, Yang MH, Wang YZ, Zhao SS, Tan WB, Chen CF, et al. Investigation of ticks and application of DNA barcode in Junggar Basin, Xinjiang. J Shihezi Univ. (Nat Sci). 2019;5:575–9 (In Chinese with English abstract).
11. Jia N, Wang J, Shi W, Du L, Sun Y, Zhan W, et al. Large-scale comparative analyses of tick genomes elucidate their genetic diversity and vector capacities. Cell. 2020;182:1328–40.
